# Unveiling the gut-liver axis: the behind-the-scenes “manipulator” of human immune function

**DOI:** 10.3389/fimmu.2025.1638197

**Published:** 2025-11-10

**Authors:** Peizhe Li, Yu Wang, Yanan Dong, Xin Zhang

**Affiliations:** Department of Acupuncture and Massage, Changchun University of Chinese Medicine, Jilin, China

**Keywords:** gut-liver axis, immunoregulation, gut microbiota, bile acids, immune function

## Abstract

The “gut-liver axis” enables bidirectional immunoregulation between the intestine and the liver through the portal venous circulation, bile acid metabolism, and the neuro-lymphatic network. This paper reviews its physiological pathways (vascular, biliary, neural, and lymphatic), immunomodulatory mechanisms (interaction of innate/adaptive immune cells, balance between inflammation and tolerance), and associations with diseases such as PSC, MAFLD, and IBD. Metabolites of gut microbiota activate immune cell receptors to regulate the differentiation of Tregs, while cytokines (such as IL-6) and chemokines (such as CCR9) drive the synergy of gut-liver immunity. In pathological conditions, dysbiosis, endotoxin translocation, and bile acid metabolic disorders trigger immunological dysregulation through this axis. Strategies such as targeted fecal microbiota transplantation and bile acid receptor (FXR) agonists show clinical potential. This paper systematically elaborates on the physiological and immunoregulatory mechanisms of the “gut-liver axis”, explores the associations between its abnormalities and immune diseases, as well as the prospects of translational medicine. It is proposed that future research should deepen the analysis of single-cell interactions, conduct personalized interventions, and establish a new paradigm of “gut-liver axis medicine” to provide cross-organ solutions for the precise prevention and control of immune-related diseases.

## 1 Introduction

In recent years, the unexpected emergence of the COVID-19 pandemic and the rapid spread of monkeypox outbreaks in 2022 and 2024 have increased populations’ susceptibility to infectious diseases and severe complications, posing unexpected challenges to public health (1). The World Health Organization has also warned that building a strong immune barrier is crucial in the face of upcoming “X diseases” (2). Since immune responses vary due to individual innate and genetic factors, and changes in T-cell and B-cell populations may lead to chronic low-grade inflammation, this can cause increased cytokines in the body, gut microbiota disorders, and liver immune dysfunction (3). Such changes exacerbate the decline in the body’s immune system function, forming a vicious cycle.

Traditionally, immunology has focused on single organs, but growing evidence indicates that the homeostasis of immune function relies on the synergistic action of the ‘gut-liver axis’ formed by the intestine and liver through portal venous circulation, bile acid metabolism, and neuro-lymphatic networks. The “gut-liver axis” refers to the bidirectional relationship between the intestine and its microbiota and the liver, which is generated by the integration of signals from dietary, genetic, and environmental factors. This process primarily involves two aspects: Through the portal venous circulation, the liver serves as the primary recipient of gut-derived metabolites and microbial products. The liver secretes products into the intestine via the biliary system (4).

**Figure 1:**
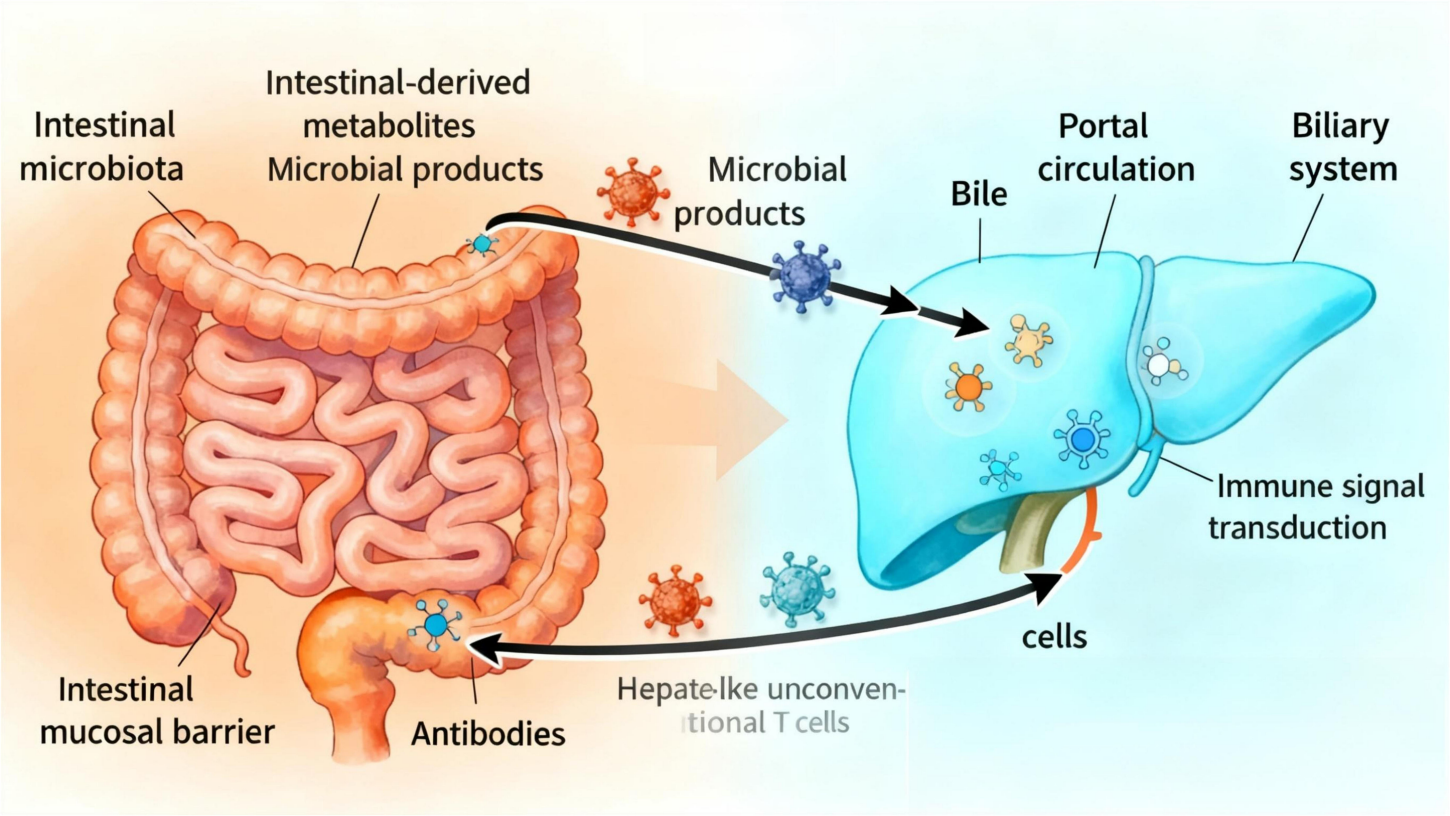
Gut-liver axis schematic.

Thus, the gut-liver axis is established by the vascular pathway of the portal vein (which directly delivers intestinal-derived products to the liver) and the hepatic feedback pathway by which bile and antibodies enter the intestine (5). Previous studies have shown (6) that the liver is the first organ with unique anatomical and immune sites capable of directly or indirectly activating lymphocytes, endowing it with specialized immunological properties. The intestine, as a critical immune organ in the human body, often plays a central role in systemic immune function. One experiment demonstrated (4) that the “gut-liver axis” is enriched with various innate immune cells, including innate-like unconventional T cells and adaptive T cells. These cells are believed to participate in maintaining tolerance to gut-derived antigens while enabling effective immune responses against microbes. Interestingly, the transmission of immune signals and activation of functions in the body depend on the normal operation of the gut-liver axis. The interdependence between the intestine and liver explains why disruption of the intestinal barrier can lead to increased portal venous flow of bacteria or their products into the liver, thereby triggering or worsening a range of diseases. Specifically, dysfunction of the gut-liver axis impairs intestinal mucosal barrier function, disrupts gut microbiota, disturbs liver metabolism, and imbalances immune system function, ultimately leading to disease development (**Figure 1**).

With the development of single-cell sequencing, organoid technology, and metagenomics, the academic community has been able to analyze the immunoregulatory mechanisms of the “gut-liver axis” at the levels of cell subsets, molecular interactions, and microbial community functions. This paper systematically reviews the anatomical basis, immunoregulatory mechanisms, and roles in diseases of the “gut-liver axis,” aiming to address the following core questions: Which pathways does the “gut-liver axis” use to achieve immune signal transmission; How do gut microbes influence the phenotypes of immune cells through this axis; and Can targeting the “gut-liver axis” provide new strategies for the treatment of immune diseases?

Elucidating the immunoregulatory mechanisms of the “gut-liver axis” not only deepens the understanding of how “organ crosstalk determines immune function,” but also holds promise for promoting the establishment of a “gut-liver dual-organ targeting” diagnostic and therapeutic paradigm. This could provide interdisciplinary solutions for intervening in immune diseases at their source.

## 2 Physiological mechanism of the gut-liver axis

Since Marshall proposed the concept of the “gut-liver axis” in 1998 (7), research on the relationship between intestinal and liver diseases has attracted extensive attention. The liver acts as a mediator of systemic and local innate and adaptive immunity and serves as a key site for immunomodulation (8). Originating from the same germ layer as the intestine, the liver is closely connected to the intestine through the portal venous circulation, bile acid metabolism, and immune signaling pathways, forming an interdependent and synergistic functional axis named the “gut-liver axis”.

### 2.1 Physiological connections of the gut-liver axis

#### 2.1.1 Vascular connections

The liver’s primary blood supply originates from the intestine (9), with the portal vein delivering 75–80% of hepatic blood flow, predominantly drained from the intestines and other visceral structures. Portal venous blood merges with hepatic arterial blood as it enters the hepatic sinusoids. The hepatic sinusoidal endothelium is formed by liver sinusoidal endothelial cells (LSECs), which exhibit a unique structure: their fenestrations (pore diameters of ~150–200 nm) lack a basal membrane, conferring high permeability (10). Additionally, the liver’s specialized anatomical vascular system enables continuous communication among immune cells, LSECs, and hepatocytes. Notably, the low-pressure blood flow and fenestrated endothelium in the liver facilitate interactions between immune cells and hepatocytes (11).

**Figure 2:**
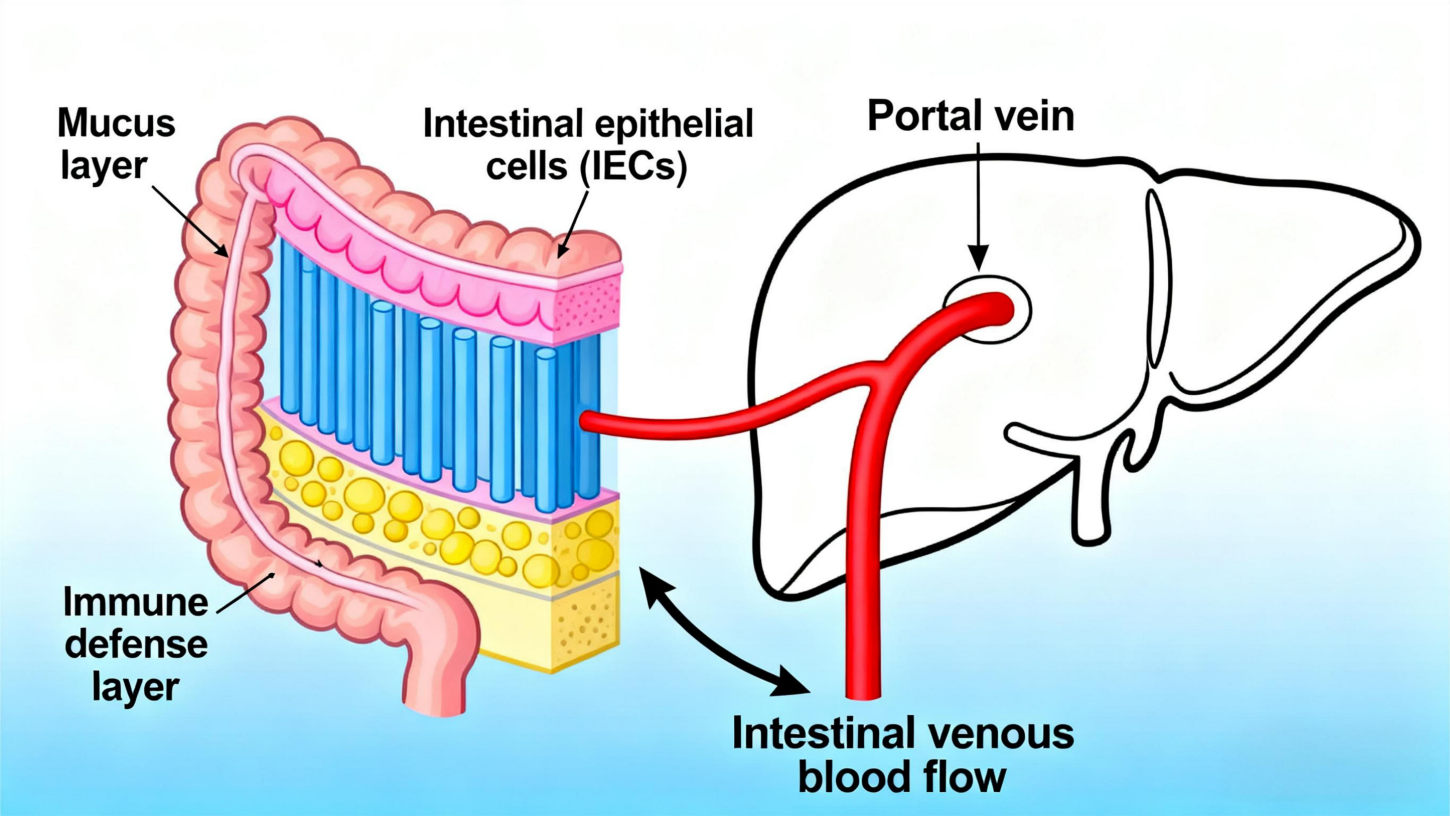
Gut-liver axis vascular connections schematic.

Meanwhile, the intricate reciprocal relationship between the liver and intestine is established via the portal vein, a critical conduit for transporting substances from the intestine to the liver. This vascular pathway promotes bidirectional communication between the gastrointestinal tract and liver, supported by a multi-layered intestinal barrier: the superficial mucus layer, the intermediate physical barrier composed of intestinal epithelial cells (IECs), and the inner immune defense layer (12). As intestinal venous blood drains into the portal vein, the liver becomes the first organ to receive gut microbiota-derived products and metabolites (**Figure 2**), indicating that microbial metabolites migrate to the liver via the portal vein to influence hepatic gene expression and physiological processes (13). In a diet-induced obesity mouse model with gut dysbiosis, Heetanshi Jain et al. investigated the biodistribution of bEVs along the gut-liver portal vein-liver axis and found that extracellular vesicles (EVs) secreted by all living organisms (including bacteria) can cross the intestinal mucosal barrier into the systemic circulation and accumulate in the liver (14). Thus, as a hub for interactions between the intestine and other tissues, the liver integrates signals from the gastrointestinal tract and adipose tissue to regulate metabolism of carbohydrates, lipids, and amino acids (15).

#### 2.1.2 Biliary system

Bile is produced in hepatocytes and gradually converges through intrahepatic bile ducts into extrahepatic ducts, including the left and right hepatic ducts, common hepatic duct, and common bile duct. The common bile duct eventually merges with the pancreatic duct and opens into the duodenal papilla to discharge bile into the duodenum (16). Meanwhile, bile excreted into the intestine via the biliary tract is reabsorbed, influencing biliary-associated bacteria and altering the composition of the gut microbiota. Studies have shown (17) that in cholestasis models, the absence of intestinal bile disrupts gut microbiota and intestinal metabolism. Bile aids fat digestion and absorption in the intestine by emulsifying fats into tiny particles, increasing their contact area with lipases to promote decomposition and absorption. An experiment on mice with cholestatic hepatic fibrosis suggested (18) that certain gut microbes are closely associated with liver metabolites transported via the biliary tract from the intestine.

**Figure 3:**
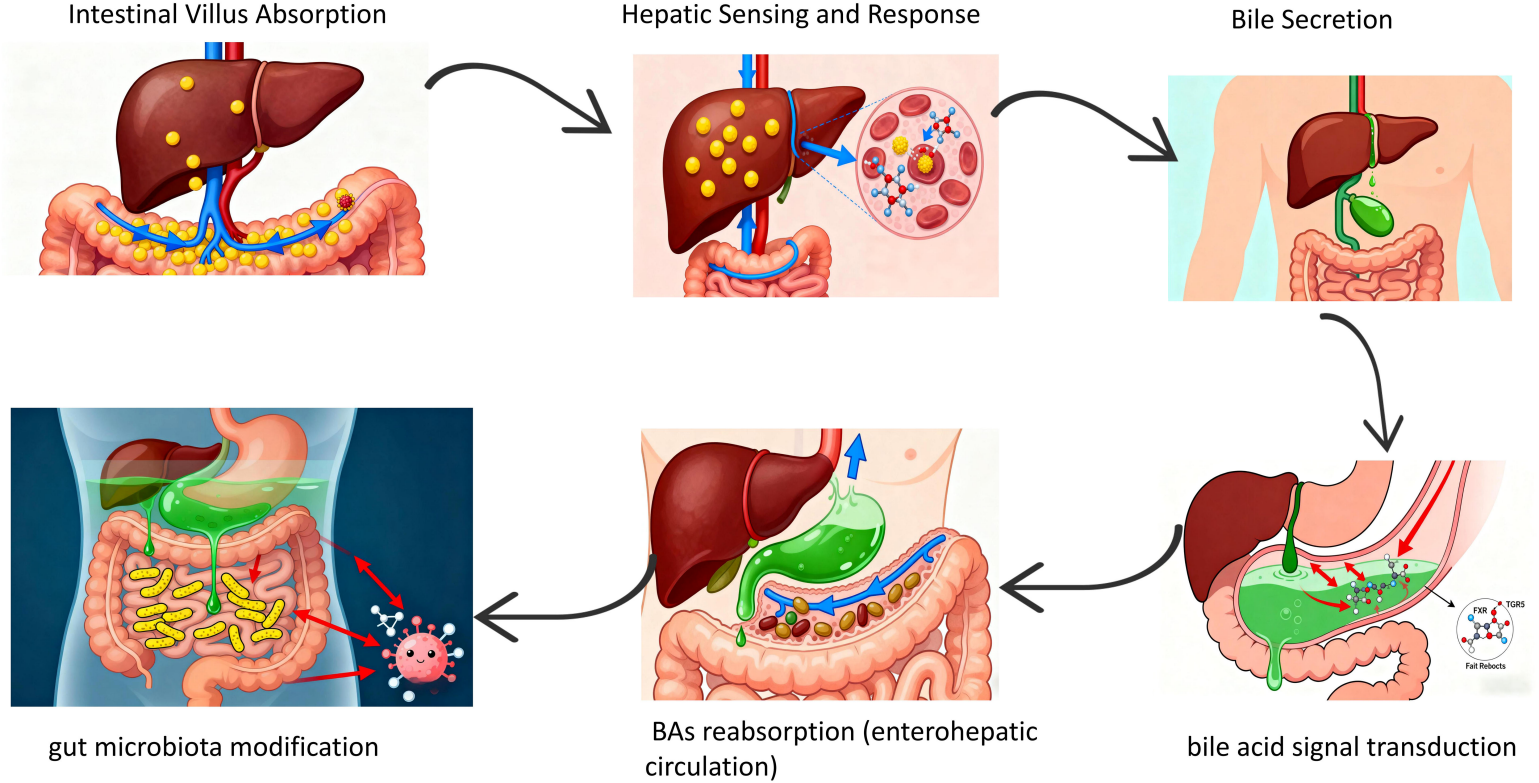
Biliary duct system.

The liver receives blood enriched in nutrients, dietary and microbial antigens directly from the intestine via the hepatic portal vein. This anatomical connection enables the liver to sense and respond to macronutrients, microbial metabolites, toxins, and other signaling molecules derived from the gastrointestinal tract. Conversely, the liver secretes bile and additional bioactive factors into the duodenum of the small intestine, which not only facilitates the digestion and absorption of dietary fats but also transduces signals through bile acid (BAs)-specific receptors, including farnesoid X receptor (FXR) and Takeda G protein-coupled receptor 5 (TGR5), among others (19). BAs, a critical component of bile, play a vital role in intestinal digestion and absorption of lipids and fat-soluble vitamins (20). BAs are reabsorbed in the ileum via active transport proteins and returned to the liver through the portal vein—a process termed the “enterohepatic circulation” (21), which is essential for maintaining stable bile composition and normal bile secretion. These BAs are stored in the gallbladder or secreted into the duodenum to facilitate lipid absorption. Most are reabsorbed in the terminal ileum via active transport, while partially modified secondary BAs generate various isomers regulated by gut bacteria in the ileum and colon. These isomers may potentially regulate immune, inflammatory, and endocrine homeostasis (22–24) (**Figure 3**).

#### 2.1.3 Neural regulation

Anatomically, the liver is innervated by both the sympathetic and parasympathetic nervous systems (25). The intestine, containing 200 million to 600 million neuronal cell bodies, is the most densely innervated peripheral organ in the body. Within the intestinal wall, afferent nerve fibers (NG neurons and DRG neurons), sympathetic fibers, parasympathetic nerves, and the enteric nervous system (ENS) form a complex neural network (26). Typically, parasympathetic pathways in the gastrointestinal tract are excitatory (27), primarily acting via the vagus nerve (innervating the esophagus, stomach, pancreas, and upper large intestine) and pelvic nerves (innervating the lower large intestine, rectum, and anus). Studies have shown (28) that the autonomic nervous system can stimulate cholecystokinin release via intestinal fat stimulation, thereby enhancing intestinal peristalsis. Thus, both the liver and intestine receive dual innervation from sympathetic and parasympathetic nerves: Sympathetic activation constricts hepatic blood vessels, reduces hepatic blood flow, and inhibits intestinal peristalsis and digestive juice secretion. Parasympathetic activation promotes hepatic blood circulation and bile secretion, while enhancing intestinal peristalsis and digestive juice secretion to facilitate digestion and absorption.

Neural reflexes also connect the liver and intestine. For example, intestinal irritation can trigger neural reflexes that alter hepatic bile secretion, while liver pathologies may influence intestinal function via neural reflexes, leading to symptoms such as poor digestion and malabsorption.

#### 2.1.4 Lymphatic circulation

**Figure 4:**
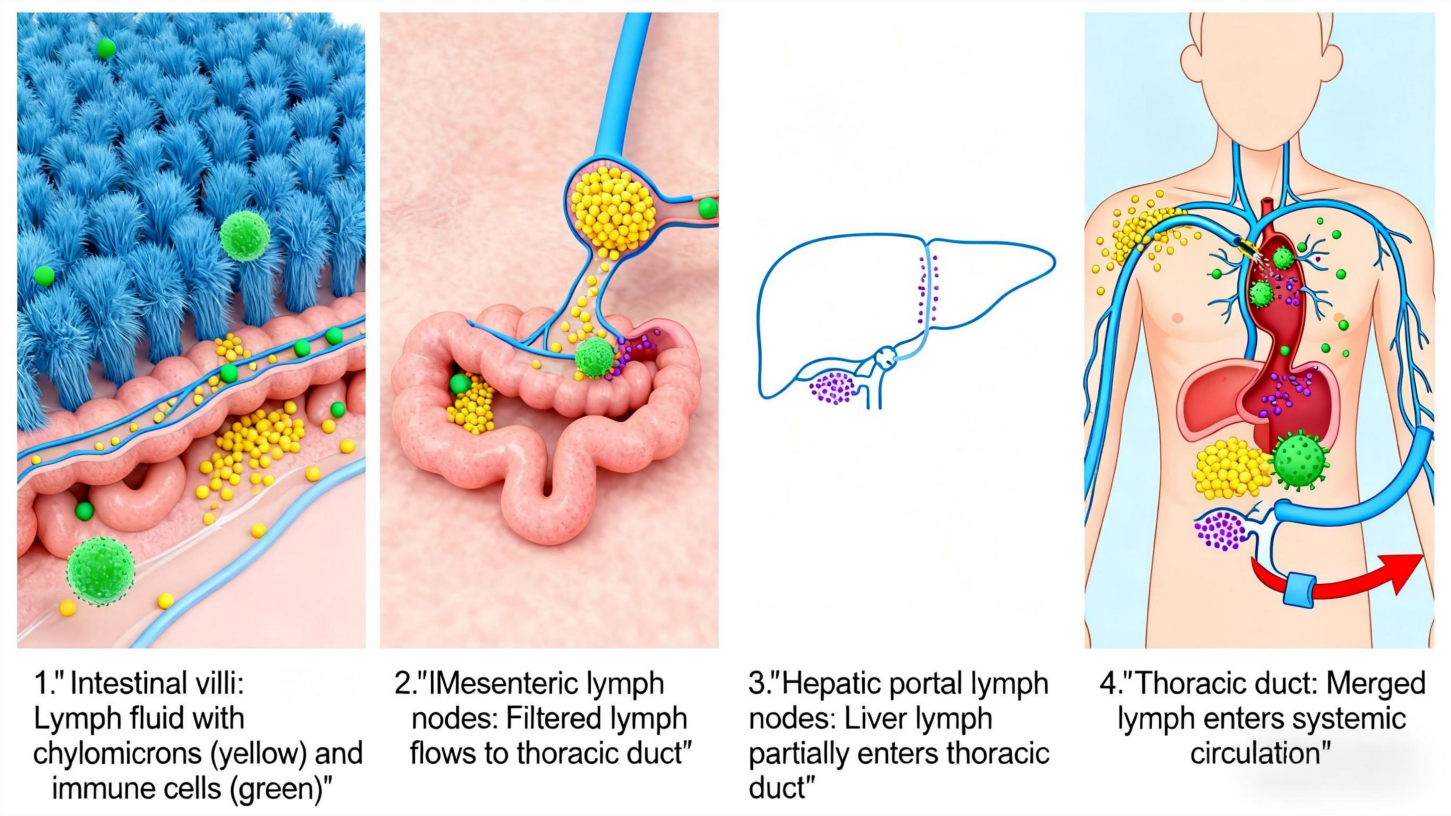
Lymphatic circulation schematic.

In the intestine, lymphatic fluid is rich in diet-derived lipids incorporated into chylomicrons and gut-specific immune cells. Intestinal lymphatic vessels are therefore critical for systemic delivery of dietary lipids and metabolic regulation (29). Metabolites from dietary compounds and the gut microbiome can enter the lymphatic system to modulate distal organs like the liver (30). The hepatic and intestinal lymphatic systems are interconnected: Intestinal lymph contains substances such as fat particles and immunoglobulins, which converge through mesenteric lymph nodes into the thoracic duct before entering the bloodstream. Hepatic lymph drains via hepatic portal lymph nodes, with some flowing into the thoracic duct and the rest directly entering the bloodstream (**Figure 4**).

## 3 Overview of systemic immunity

### 3.1 Composition of immune functions

**Figure 5:**
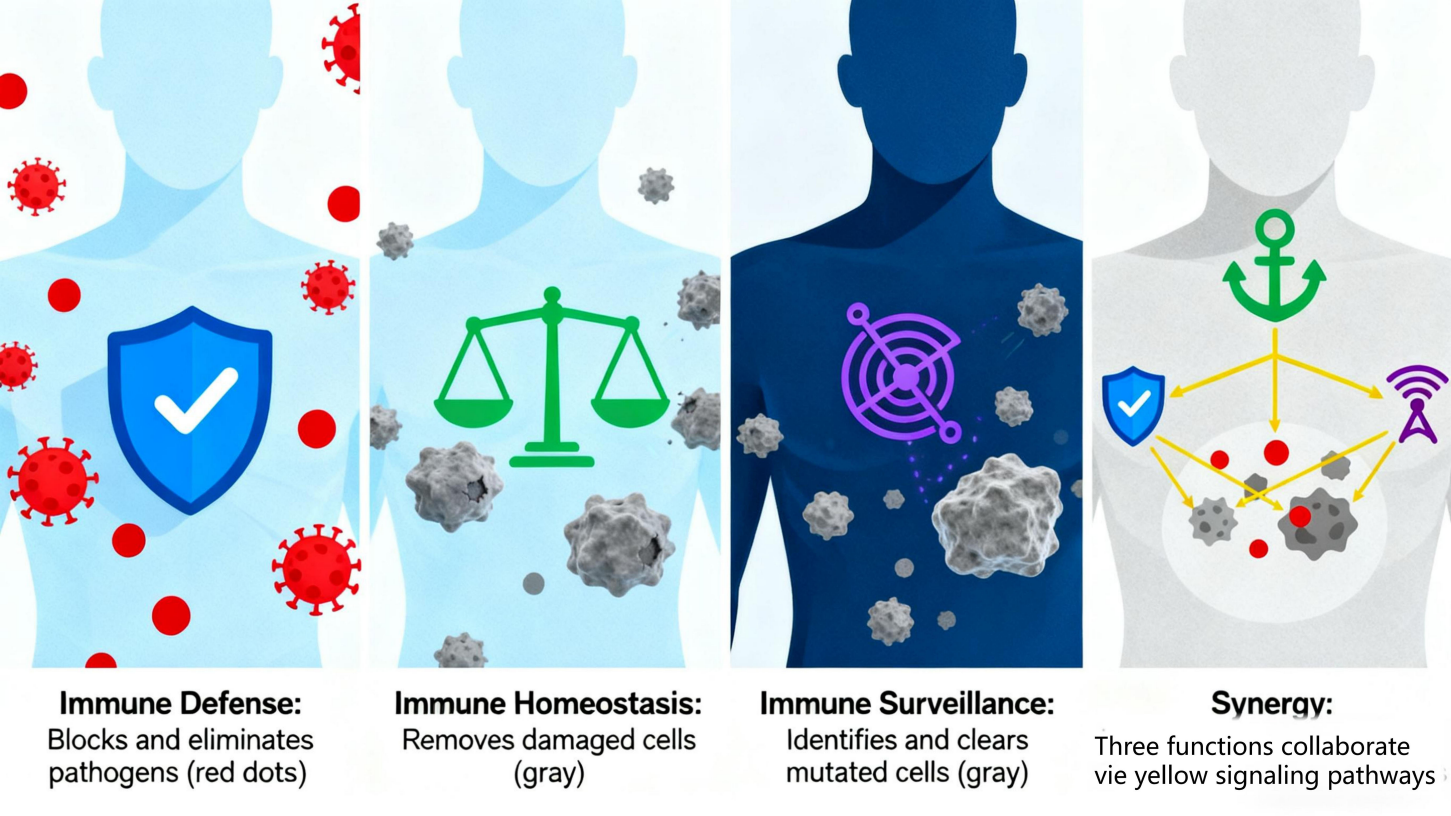
The composition of the body's immune function.

The immune function of the human body is primarily composed of three major functions: immune defense, immune surveillance, and immune homeostasis. These functions are interconnected and mutually influential, collectively maintaining the body’s health. Immune defense serves as the foundation, preventing the invasion of external pathogens and eliminating infiltrated pathogens and other harmful substances. Immune homeostasis acts as a critical safeguard, maintaining internal environmental stability by continuously removing aged, damaged, or denatured self-cells and identifying/eliminating antigenic foreign substances. Immune surveillance is pivotal, enabling the timely detection and elimination of mutated cells and virus-infected cells in the body to prevent tumorigenesis and persistent viral infections. Immune surveillance collaborates with defense and homeostasis: Defense and homeostasis clear pathogens and harmful substances that may induce cell mutation, reducing tumorigenic triggers. Surveillance identifies and eliminates abnormal cells generated by homeostatic dysfunction and virus-infected cells not fully cleared by defense, preventing their progression to tumors or persistent infections. The three immune functions work in close coordination to form a complete defense system. Abnormalities in any function may disrupt others, leading to immune dysfunction and various diseases (**Figure 5**).

#### 3.1.1 Immune defense

Immune defense is an immunoprotective function that defends against foreign pathogen invasion and clears external antigenic substances, primarily accomplished through innate immunity and adaptive immunity (31).

Innate immunity (first line of defense) includes physical barriers (skin, mucosa) and immune cells (phagocytes, natural killer cells), rapidly recognizing and eliminating pathogens. Adaptive immunity (built upon innate immunity) is mediated by T/B cells, generating specific immune responses against target pathogens to produce antibodies and memory cells for precise clearance and long-term protection. Dysregulation of immune defense triggers diseases: Overactivity induces hypersensitivity reactions (e.g., allergic rhinitis, asthma) (32). Hypoactivity leads to immunodeficiency diseases (e.g., AIDS, with increased susceptibility to opportunistic infections due to immune system damage).

#### 3.1.2 Immune surveillance

Immune surveillance is the function of identifying and eliminating mutated cells and virus-infected cells, primarily relying on immune cells like natural killer cells and cytotoxic T cells (33, 34). These cells recognize abnormal antigens on tumor/virus-infected cells, inducing apoptosis via cytotoxic substances (perforin, granzyme) to prevent tumor development and viral persistence. Reduced surveillance capacity allows undetected mutated cells to drive tumorigenesis and impairs clearance of virus-infected cells, leading to chronic infections (35).

#### 3.1.3 Immune homeostasis

Immune homeostasis refers to the maintenance of internal environmental stability by clearing aged, damaged, or denatured self-cells, achieved through immune recognition/tolerance of self-components and phagocytosis of senescent cells (36). Phagocytes (e.g., macrophages) engulf and metabolize aged/dead cells to sustain cellular renewal (37). The immune system also regulates immune cell activity/numbers to maintain appropriate immune responses and avoid autoimmunity. Homeostatic dysfunction causes the immune system to mistakenly attack self-tissues, leading to inflammation and tissue damage in autoimmune diseases (e.g. rheumatoid arthritis, systemic lupus erythematosus).

### 3.2 Composition of the immune system

The human immune system is composed of immune organs, immune cells, and immune molecules, playing a critical role in maintaining normal bodily functions.

#### 3.2.1 Immune organs

**Figure 6:**
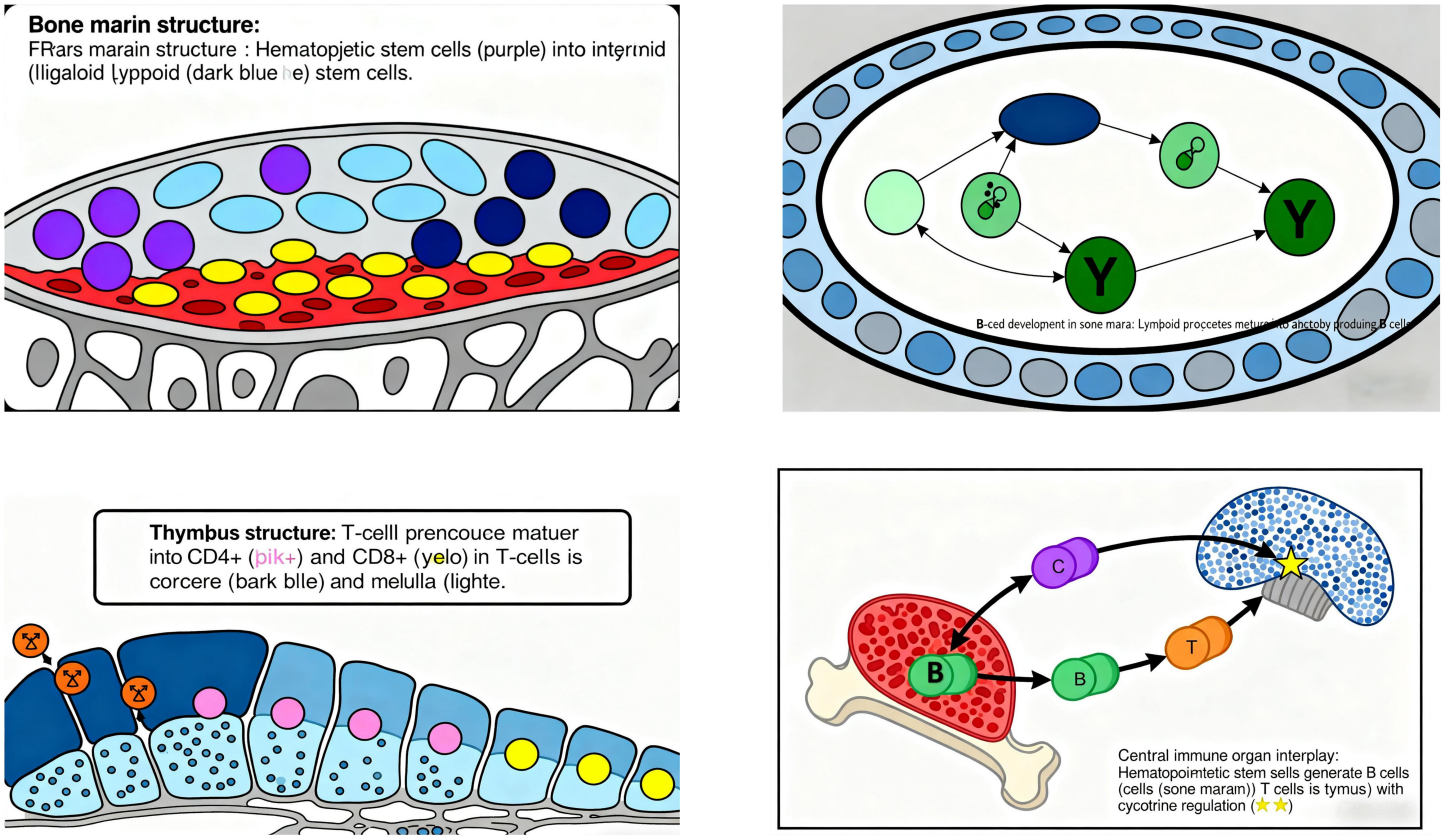
Brief schematic of immune organs.

Central immune organs include the bone marrow and thymus: Bone marrow is the site for the generation, differentiation, and maturation of various immune cells, serving as the primary locus for B-cell development. It also produces cytokines involved in immunoregulation. Before and after birth, the bone marrow primarily functions in hematopoiesis, generating precursor cells for all immune cells (including T-cell progenitors). Multipotent stem cells in the bone marrow differentiate into myeloid and lymphoid stem cells: Myeloid stem cells give rise to erythrocytes, monocytes, granulocytes, and megakaryocytes. Lymphoid stem cells develop into B-cell and T-cell precursors, which are central to humoral and cellular immunity, respectively. During B-cell development, lymphoid progenitors mature as they migrate toward the center of the bone marrow cavity (38) (**Figure 6**).

Thymus is the site of T-cell differentiation and maturation (39), consisting of the cortex and medulla. Composed of thymic lobules, it contains thymic stromal cells (epithelial cells, macrophages, dendritic cells) and extracellular matrix, forming a microenvironment for T-cell development. Stromal cells influence T-cell proliferation, differentiation, and selection via immune molecule secretion or cell-cell contact, while the extracellular matrix maintains cellular function and promotes cell interaction/maturation (40). T-cell development begins with precursor T cells from the bone marrow entering the thymus, where they undergo selective differentiation upon contact with stromal cells. Approximately 5% of thymocytes mature into functional T-cell subsets, which then migrate from the medulla to the bloodstream (41). Key checkpoints include: Positive selection: Thymocytes with TCRs binding self-MHC molecules survive, establishing MHC restriction in antigen recognition. Negative selection: Self-reactive T cells expressing TCRs against self-antigens undergo apoptosis or anergy, ensuring immune tolerance to self-antigens (38).

Peripheral immune organs include lymph nodes, spleen, and mucosa-associated lymphoid tissue (MALT): Lymph nodes (LNs) are critical for systemic immune surveillance, serving as primary sites for lymph filtration, antigen recognition, and immune cell activation, as well as habitats for T/B-cell residence and proliferation (42). LNs compartmentalize immune cell types to facilitate antigen exposure, concentrating antigens and bridging antigen-presenting cells (e.g., DCs, B cells) with adaptive immune cells (circulating B/T lymphocytes) to coordinate effective responses. In healthy LNs, B cells cluster in lymphoid follicles, while T cells localize to the deeper paracortical regions (43). Immune dysfunction triggers lymphatic system remodeling (e.g., lymphangiogenesis, altered fluid transport, LN morphological changes), promoting inflammation and chronic inflammatory states (44).

**Figure 7:**
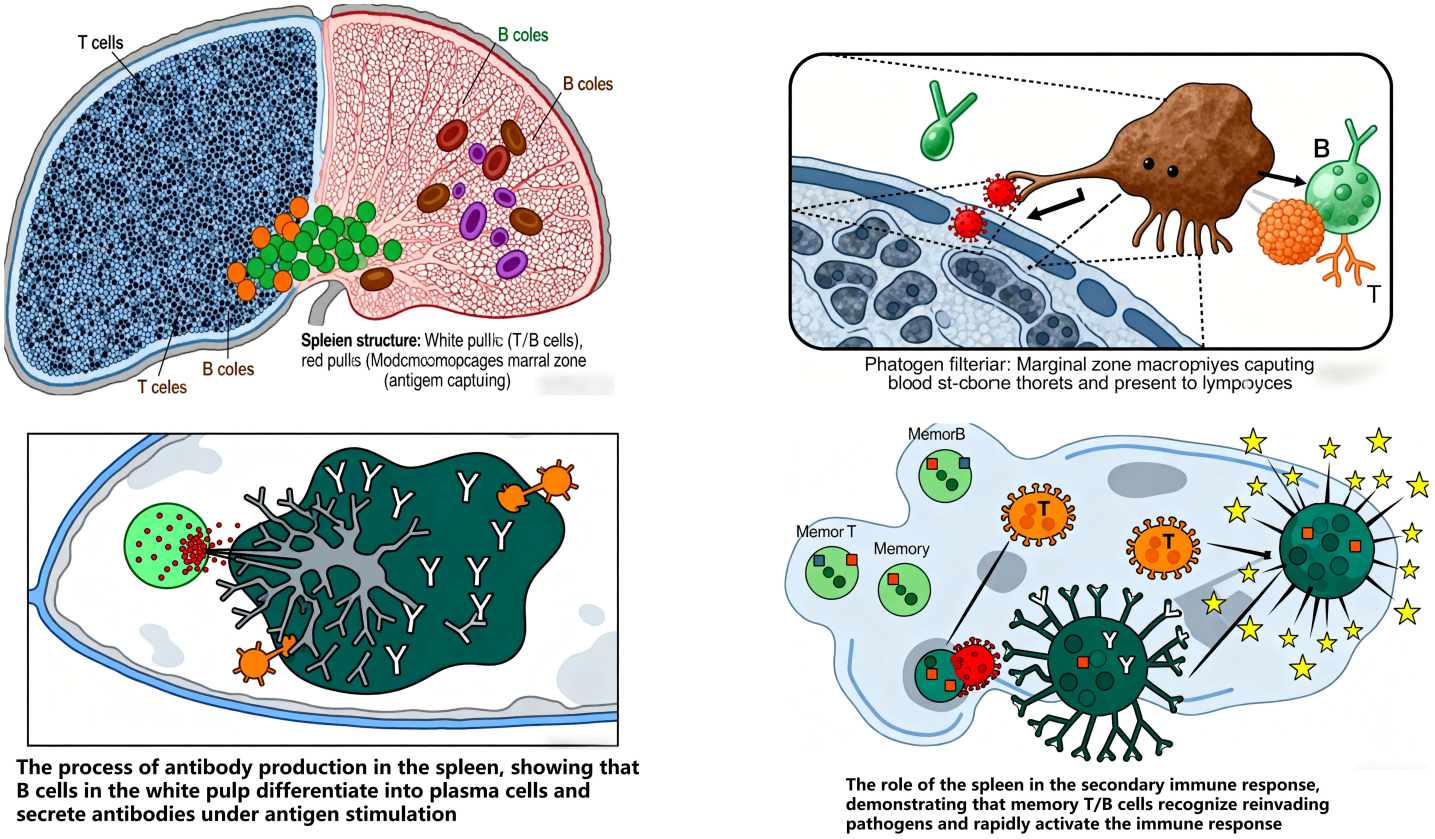
Overview of splenic immune function.

Spleen acts as an immune gatekeeper, initiating and maintaining responses against blood-borne pathogens. It filters pathogens and aged cells, and is central to blood-borne antibody production (45). The spleen index, a key indicator of immune function, reflects lymphoid tissue hyperplasia and lymphocyte activation/proliferation following antigen stimulation, with increased organ weight indicating enhanced immunity (46). Its parenchyma is divided into white pulp, red pulp, and marginal zone: White pulp (dense lymphocytes) is the primary site of specific immune responses. Marginal zone (predominantly B cells with abundant macrophages) captures antigens and initiates immune responses (47, 48). Memory T/B cells are predominantly generated in the spleen, underscoring its role in secondary immune responses (49) (**Figure 7**).

MALT is widely distributed in the respiratory, gastrointestinal, and urogenital tracts, serving as the first line of defense against pathogens and mediating local immune responses. Following viral infection, the mucosal surface—the first interface with external antigens—triggers mucosal immunity via the mucosal immune system (MIS), involving innate responses in epithelial tissues, local inflammation, and adaptive responses in MALT (50). Mucosal infections typically activate antigen-specific B cells in regional MALT (51). For example, the intestinal mucosal barrier—comprising epithelial, immune, biological, and chemical barriers—balances nutrient absorption with defense against harmful molecules/microbes. These interdependent barriers form a complex protective network (52). The intestinal mucosal immune system, known as gut-associated lymphoid tissue (GALT) includes: Inductive sites (structured follicles), Capture and process antigens for presentation to immune cells. Effector sites (diffuse lymphoid tissue in the lamina propria): Where plasma cells and sensitized lymphocytes migrate via homing mechanisms to exert immune functions (53).

#### 3.2.2 Immune cells

Innate immune cells are typically activated during the early stages of viral infection to prevent viral replication, dissemination, and clearance, while also facilitating the development of adaptive immune responses. These primarily include macrophages, dendritic cells (DCs), neutrophils, and natural killer (NK) cells (54) Macrophages are essential immune cells that phagocytose and kill pathogens, present antigens, and secrete cytokines, playing a critical role in inflammation and tissue homeostasis. They contribute to immune surveillance, host protection, and tissue-specific homeostatic functions (55). Studies have shown (56) that chronic inflammatory diseases such as atherosclerosis, diabetes, cardiovascular disease, and liver dysfunction are closely associated with impaired macrophage metabolism. Additionally, macrophages exhibit high plasticity, regulating host immune responses through differentiation and polarization in different tissue microenvironments (57). Dendritic cells (DCs) are key antigen-presenting cells that recognize, process, and present antigens to T cells, bridging innate and adaptive immunity (58). DCs detect pathogen-associated molecular patterns (PAMPs, e.g., from bacteria, viruses, fungi, parasites) and damage-associated molecular patterns (DAMPs) via pattern-recognition receptors (PRRs), including Toll-like receptors (TLRs), C-type lectin receptors (CLRs), NOD-like receptors (NLRs), and RIG-I-like receptors (RLRs). This initiates immune responses to combat infection and repair tissue damage (59). Neutrophils, the most abundant white blood cells in the bloodstream (comprising 60–70% of all leukocytes in human blood) (60), serve as the first responders of the innate immune system. Their critical role in combating invading pathogens is evidenced by the severe susceptibility to infections in neutropenic patients. Neutrophils rapidly migrate to infection sites to clear pathogens through phagocytosis and release of bactericidal substances (61).Natural killer (NK) cells, key components of the liver’s innate immune system, account for 30–50% of intrahepatic lymphocytes (62). They recognize and kill virus-infected cells and tumor cells, exerting important immune surveillance functions in innate immunity. Research indicates (63) that NK cells participate in multiple non-infectious inflammatory diseases, potentially exerting dual anti-inflammatory and pro-inflammatory effects through changes in subset populations, activation/inhibition of receptors, regulation of inflammatory cytokine secretion, and cytotoxic activity.

Adaptive immune cells, primarily including T cells and B cells, play a critical role in maintaining bodily functions by resisting pathogen invasion and preventing various diseases. Abnormalities in these cells—triggered by factors such as inflammation or tumors—can cause immune imbalance and threaten health. As key components of adaptive immunity, T and B cells are essential for maintaining immune function; numerical and/or functional abnormalities in response to stimuli can lead to immune imbalance and a cascade of pathological changes (64). T lymphocytes are classified into CD4+ and CD8+ subsets based on cell surface antigens. Imbalance between CD4+/CD8+ cells is a major cause of immune dysfunction. Functionally, T cells are divided into cytotoxic T cells, helper T cells (Th), and regulatory T cells (Treg). Th cells (marked by CD4 expression) further differentiate into Th1 and Th2 subsets based on cytokine responses and secretion profiles, each exerting distinct immunological functions (65): Helper T cells assist B cells in antibody production and activate cytotoxic T cells. Cytotoxic T cells specifically kill target cells. Regulatory Tregs maintain immune tolerance through immunomodulation. Recent evidence (66) indicates that B cells represent a critical subset of adaptive immune cells in the tumor microenvironment (TME), acting as influential and multifunctional contributors to antitumor responses. Upon antigen stimulation, B cells differentiate into plasma cells to produce antibodies, mediating humoral immune responses. Current research (67) highlights regulatory B cells (Bregs) and plasma cells as key players in the progression of hematological malignancies and immunoregulation. These cells exhibit dual roles: enhancing antitumor immunity via antigen presentation and antibody production, while promoting immune evasion through secretion of immunosuppressive cytokines (e.g., IL-10).

#### 3.2.3 Immune molecules

Immune molecules refer to various biomolecules that play critical roles in immune responses, primarily including immunoglobulins, complement, and cytokines. Immunoglobulins (Igs) are large Y-shaped proteins secreted by plasma cells, used by the immune system to identify and neutralize foreign antigens. Distributed predominantly in serum, they also exist in tissue fluid, exocrine secretions, and on certain cell membranes, categorized into five classes: IgG, IgA, IgM, IgD, and IgE. As immune enhancers, Igs effectively inhibit pathogen binding to target cells, form antigen-antibody complexes to clear pathogens, and enhance macrophage function and overall immunity (68). Cytokines are small proteins secreted by immune cells and certain non-immune cells, regulating immune cell growth, differentiation, and function while mediating inflammatory responses. They modulate immune cell development, activation, and proliferation—for example, IL-12 promotes Th1 cell differentiation, while IL-10 inhibits macrophage activation and cytokine secretion, thus controlling the intensity and direction of immune responses. The complement system, a vital component of immune defense, rapidly and efficiently kills pathogens upon recognition (69). Present in serum and tissue fluid, it is activated via classical, alternative, and lectin pathways to exert functions such as bacteriolysis, lysis of virus-infected cells, opsonization, and inflammation mediation. Small fragments generated during complement activation (e.g., C3a, C5a) act as inflammatory mediators: they recruit neutrophils and monocytes to inflamed sites, enhance inflammatory responses, and induce mast cells/basophils to release histamine, causing vasodilation and increased vascular permeability. These effects facilitate the recruitment of immune cells and molecules to infection sites for defense.

## 4 Mechanisms of the gut-liver axis on immune function

### 4.1 Regulatory role of gut microbiota

**Figure 8:**
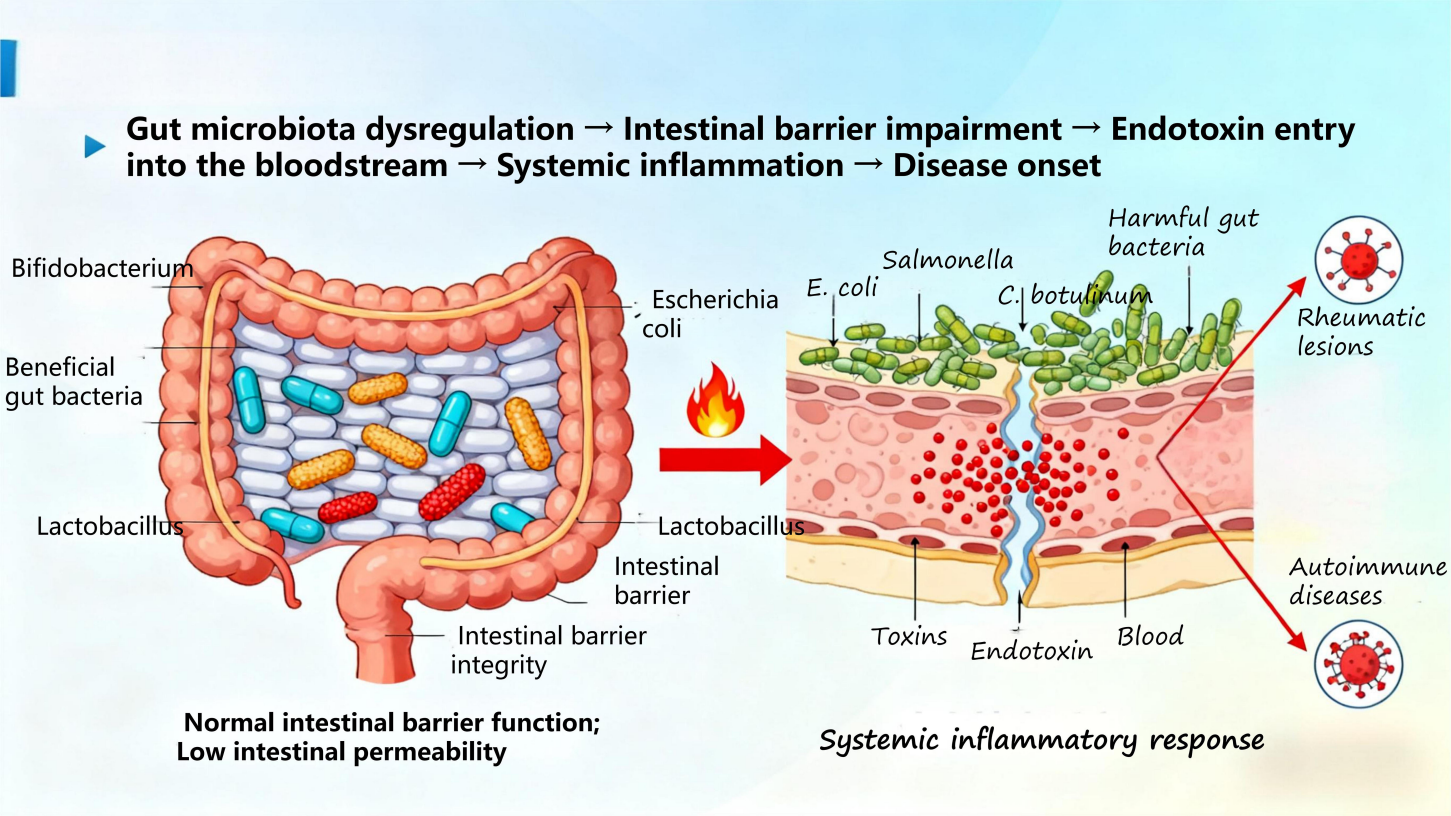
Simplified diagram of gut microbiota regulating immunity.

The development of diseases is closely linked to the disruption of bodily homeostasis. In recent years, extensive research has shown (70) that gut microbiome dysregulation participates in all stages of disease development and prognosis by influencing the host immune system and metabolism. Composed of bacteria, fungi, and viruses, the gut microbiome plays a vital role in maintaining the delicate balance of human health (71). Through Mendelian randomization analysis, Hao Sha et al. (72) demonstrated that gut microbiota is associated with autoimmune diseases (ADs), characterized by reduced beneficial bacteria, increased harmful bacteria, and decreased diversity. This imbalance impairs the intestinal barrier, increases permeability, and allows endotoxins to enter the bloodstream, triggering systemic inflammatory responses and ultimately leading to disease onset (**Figure 8**).

#### 4.1.1 Interaction with immune cells

Specific molecular motifs are expressed on the surface of gut microbiota, and lipopolysaccharide (LPS) derived from these motifs is classified as either pathogen-associated molecular patterns (PAMPs) or damage-associated molecular patterns (DAMPs)—molecules that exert a pivotal role in the pathogenesis of various liver diseases. Alterations in microbiome composition and/or increased intestinal permeability facilitate microbial translocation into the portal venous circulation, thereby enabling direct migration into the liver (73). Recent investigations have demonstrated (74) that LPS-mediated signal transduction is implicated in the pathogenesis of chronic inflammation and carcinogenesis, suggesting that the gut microbiota modulates systemic homeostasis via the gut-liver axis.

Immune cells (e.g., macrophages, dendritic cells) within the host express pattern recognition receptors (PRRs) on their cell surfaces, which specifically recognize PAMPs and DAMPs to initiate downstream immune signaling cascades (75). Among these cascades, the TLR4/NF-κB pathway stands as one of the most critical regulators of inflammatory responses. When the intestinal barrier is compromised, intestinal LPS traverses the intestinal mucosa to enter the portal vein, where it binds to TLR4 expressed on the surface of hepatic Kupffer cells and intestinal macrophages (76). This binding event recruits the downstream adaptor molecule MyD88 and initiates the activation of the NF-κB transcription factor. Activated NF-κB translocates into the cell nucleus (77), where it orchestrates the transcription, expression, and secretion of pro-inflammatory cytokines (e.g., IL-6, TNF-α, IL-1β). Concurrently, this pathway drives the chemotactic recruitment of effector immune cells (e.g., neutrophils, monocytes) to sites of inflammation, thereby triggering the host’s anti-infective immune defense response (78).

However, sustained overactivation of the TLR4/NF-κB pathway—driven by factors such as long-term LPS translocation—disrupts the host’s inflammatory-anti-inflammatory balance and induces a state of systemic chronic low-grade inflammation (79, 80). This aberrant activation not only exacerbates liver damage but also potentiates intestinal inflammatory responses (e.g., exacerbating disease severity in IBD).

Building on the aforementioned regulatory cascades, beneficial commensal bacteria in the gut produce bioactive metabolites (e.g., SCFAs) through metabolic processes. These metabolites exert a negative regulatory effect on the TLR4/NF-κB pathway, which effectively attenuates the release of pro-inflammatory cytokines and ultimately sustains intestinal and systemic immune homeostasis.

Beyond these molecular mediators, subsets of macrophages and dendritic cells (DCs) also reside within the intestinal tract. On one hand, these cells mediate antigen uptake and presentation. On the other hand, macrophages and DCs exhibit extensive functional plasticity; they collectively contribute to the maintenance of intestinal immune homeostasis by suppressing aberrant immune responses to harmless antigens and commensals, while enhancing host defense against pathogenic microorganisms (81).

Additionally, the gut microbiota modulates the differentiation trajectory of immune cells. Studies have shown (82) that certain commensal bacteria promote the differentiation of naive T cells into regulatory Tregs, which constrain excessive immune activation to preserve immune homeostasis. In contrast, some opportunistic pathogens may drive the differentiation of naive T cells into Th17 cells. Th17 cells participate in inflammatory responses and support host defense against extracellular pathogens, but their overactivation is associated with the development of autoimmune diseases.

#### 4.1.2 Involvement in immune organ development

Gut microbiota are a key factor in the development of GALT, primarily influencing the human immune system through GALT (83). For example, gut-homing T cells mainly originate from GALT and are transported to the gastrointestinal tract via gut-homing integrins; their interaction with local hormones determines the residence of immune cells in normal and damaged gastrointestinal tissues (84). Increasing evidence also indicates (85) that the pathogenesis of type 1 diabetes (T1D) is associated with complex interactions between GALT and gut microbiota. Both animal experiments and clinical studies suggest (86) that gut dysbiosis precedes the development of T1D in humans and mice, with loss of intestinal barrier integrity and low-grade intestinal inflammation observed in first-degree relatives of T1D patients at high risk of disease development. An animal study in China (87) showed that animals raised in a germ-free environment exhibit poor GALT development, smaller structures such as Peyer’s patches and mesenteric lymph nodes, and reduced numbers of immune cells. Developmental defects in germ-free animals primarily affect primary and secondary immune organs such as GALT, spleen, and thymus, along with a more developed cecum, longer villi, narrower crypts, and smaller Peyer’s patches and mesenteric lymph nodes. However, colonization of germ-free mice with microbiota from conventional mice or humans restores lymphatic system development within 3 weeks, suggesting that microbiota may be a major factor promoting postnatal maturation of the intestinal mucosal immune system. This also demonstrates that microbial exposure during infancy influences the developmental morphology and function of the immune system, with commensal bacteria colonization contributing to the development, expansion, and education of the mucosal immune system, thereby directly or indirectly affecting immune system maturation.

Microbiota can enter the bloodstream through multiple pathways to act on immune organs such as bone marrow and spleen, influencing the differentiation of hematopoietic stem cells and the production of immune cells. Metabolites of gut microbiota, such as SCFAs, are mainly produced by gut microbes during the fermentation of partially indigestible polysaccharides. As key participants in the interaction between diet, microbiota, and health, SCFAs regulate the production of immune mediators, cytokines, and chemokines, as well as the differentiation, recruitment, and activation of immune cells (e.g., neutrophils, macrophages, DCs, and T lymphocytes), thereby participating in immune system diseases and providing a certain degree of protection/damage in various diseases (88). Therefore, gut microbiota can indirectly influence the development and function of systemic immune organs through interactions with the intestinal immune system.

#### 4.1.3 Maintenance of intestinal mucosal barrier function

The gastrointestinal tract is optimized for efficient nutrient absorption while providing an effective barrier against various luminal environmental compounds. Different regulatory mechanisms in the body work together to maintain intestinal homeostasis, but alterations in these mechanisms can lead to gastrointestinal barrier dysfunction and are associated with several types of inflammation common in chronic diseases (89). Gut microbiota form a biofilm on the intestinal mucosal surface, tightly bound to intestinal epithelial cells to form a physical barrier that prevents pathogens from contacting intestinal epithelial cells and reduces the opportunity for pathogen invasion (90). Gut microbiota can metabolize substances such as antimicrobial peptides, which regulate intestinal pH, inhibit the growth of harmful bacteria, enhance the tight junctions of intestinal epithelial cells, improve the integrity of the intestinal mucosal barrier, reduce the entry of harmful substances and pathogens into the body, and thereby alleviate stimulation of the immune system (91).

#### 4.1.4 Retrograde regulation of intestinal microbiota and immunity by hepatic metabolites

**Figure 9:**
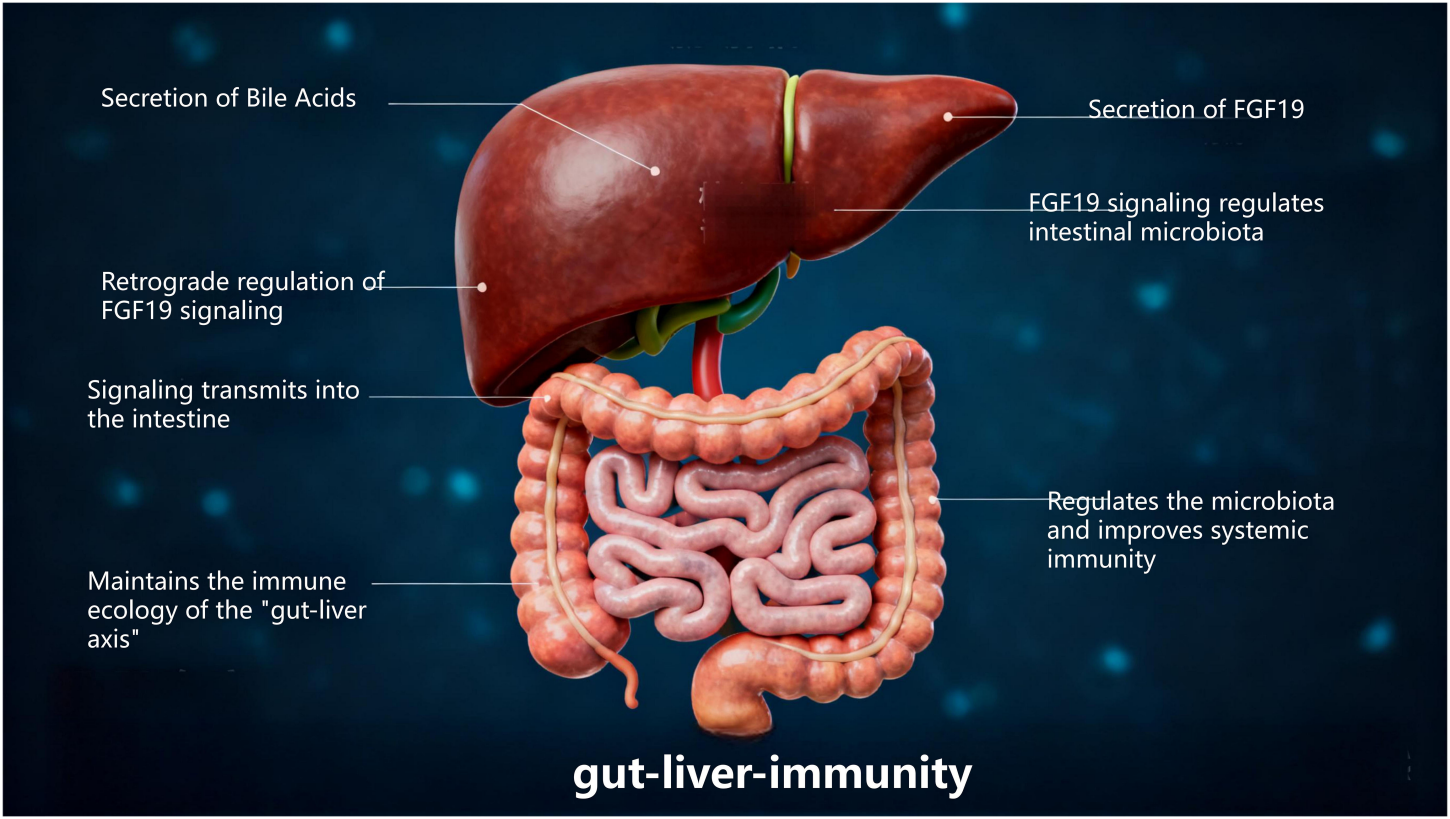
Schematic of reverse regulation of gut microbiota and immunity by liver metabolites.

As the core regulatory terminal of the “gut-liver axis”, the liver modulates the composition of intestinal microbiota in a retrograde manner and regulates intestinal as well as systemic immunity either directly or indirectly. This regulation is mediated through pathways including BAs secretion and FGF19 signaling modulation, which collectively constitutes a pivotal feedback regulatory loop for maintaining “gut-liver-immunity” homeostasis (**Figure 9**).

BAs are *de novo* synthesized by hepatocytes and excreted into the intestinal lumen via the biliary tract (92). Beyond their well-characterized role in mediating lipid digestion, BAs function as key signaling molecules that orchestrate the selective modulation of intestinal microbiota and precise regulation of immune homeostasis through two core pathways: the FXR pathway and the TGR5 pathway (93). In contrast, FGF19 indirectly contributes to the maintenance of microbiota and immune balance by exerting negative feedback control over hepatic BAs synthesis (94).

The FXR Pathway is a core regulator of basal microbiota homeostasis and immune tolerance. FXR is predominantly expressed in intestinal epithelial cells, immune cells within the terminal ileum (e.g., dendritic cells, regulatory Treg), and hepatocytes (95). Its activation occurs in a ligand-dependent manner, relying on primary BAs endogenously secreted by the liver. Following reabsorption through the brush border membrane of ileal epithelial cells, primary BAs bind to FXR on the intestinal cell surface, thereby triggering FXR-mediated signaling cascades (96, 97): On the one hand, FXR activation induces intestinal epithelial cells to secrete antimicrobial peptides (e.g., Reg3γ, Reg3β). These peptides exert specific inhibitory effects on pathogenic bacteria, limiting their over-proliferation in the intestinal niche. On the other hand, FXR regulates the expression of intestinal bile acid transporters, which maintains the steady-state concentration of BAs in the intestinal lumen. This stable BAs microenvironment supports the colonization and enrichment of beneficial bacteria microbes capable of secreting bile acid hydrolase.

In intestinal immune cells, FXR activation further modulates immune tolerance: it induces dendritic cells to secrete the IL-10 while suppressing the production of IL-12. This cytokine profile shift drives the differentiation of naive T cells toward the Treg phenotype. Treg cells then enhance intestinal immune tolerance by secreting TGF-β, which prevents excessive immune activation against intestinal commensal bacteria or food antigens, thereby mitigating the risk of immune-related disorders such as food allergy and IBD. Clinical investigations have established (98) that FXR expression in the intestinal mucosa of IBD patients is significantly down-regulated. This reduction in FXR levels leads to decreased antimicrobial peptide secretion and insufficient Treg differentiation, which in turn exacerbates intestinal microbiota dysbiosis and amplifies intestinal inflammatory responses.

The TGR5 Pathway (99), a broad-spectrum regulator of anti-Inflammatory and metabolic synergy. As a key pathway mediating the coordinated regulation of inflammation and metabolism, TGR5 exhibits a more ubiquitous distribution than FXR (100). In addition to intestinal epithelial cells and immune cells, TGR5 is also expressed in adipocytes and myocytes. Its activation depends on secondary BAs —metabolites of primary BAs generated by intestinal microbiota—forming a cascading regulatory axis defined as “hepatic BAs → intestinal microbiota metabolism → TGR5 activation”.

The biological effects of TGR5 activation are multifaceted: It promotes the secretion of glucagon-like peptide 1 (GLP-1) by intestinal L cells. GLP-1 not only maintains glucose homeostasis through regulating insulin secretion but also enhances the integrity of intestinal epithelial tight junctions. This barrier-strengthening effect reduces intestinal permeability and limits the translocation of pathogenic bacteria and LPS into the systemic circulation. Furthermore, GLP-1 inhibits excessive intestinal peristalsis, prolonging the interaction duration between BA and intestinal microbiota and thereby facilitating the metabolic transformation of BA by beneficial bacteria. In intestinal macrophages, TGR5 activation inhibits the activation of NF-κB via the cyclic adenosine monophosphate (cAMP)-protein kinase A (PKA) signaling pathway. This inhibition reduces the release of pro-inflammatory cytokines (e.g., tumor necrosis factor-α/TNF-α, IL-6), alleviating local intestinal inflammation. Concurrently, TGR5 activation promotes the polarization of macrophages toward the anti-inflammatory M2 phenotype, further amplifying the anti-inflammatory response (101).

Clinical studies have confirmed that TGR5 agonists significantly ameliorate hepatic steatosis and inflammation in patients with MAFLD. The underlying mechanism involves the inhibition of intestinal inflammation, which reduces the translocation of intestinal LPS into the bloodstream and suppresses the activation of the TLR4/NF-κB pathway in hepatic Kupffer cells.

FGF19 is a core regulator of BAs concentration homeostasis and indirect mediator of microbiota-immune balance. Secreted by intestinal epithelial cells (102), FGF19’s core biological function is to exert negative feedback control over hepatic BA synthesis. Its regulatory effects on intestinal microbiota and immunity are indirectly achieved through the maintenance of BA concentration homeostasis in the intestinal lumen:

When intestinal BA concentration is excessively high, FGF19 binds to and activates hepatic FGFR (101). This activation inhibits *de novo* BAs synthesis in hepatocytes, preventing the over-suppression of beneficial bacteria and disruption of microbiota balance by supraphysiological concentrations of BAs. When intestinal BAs concentration is abnormally low, the inhibitory effect of FGF19 on hepatic BAs synthesis weakens. The liver then resumes BAs production, ensuring that BAs concentrations remain sufficient to inhibit pathogenic bacteria and reduce microbiota translocation. This process forms a regulatory buffer axis characterized as “FGF19 → BAs concentration → microbiota composition”.

Insufficient FGF19 secretion or functional abnormalities (e.g., in the context of IBD) can lead to excessive hepatic BAs synthesis and elevated intestinal BA concentrations. This dysregulation has two detrimental consequences (103):

It impairs the integrity of the intestinal mucosal barrier, increasing the translocation of pathogenic bacteria and LPS into the systemic circulation. These translocated factors activate hepatic Kupffer cells, triggering the release of inflammatory cytokines (e.g., IL-6, TNF-α) and forming a “intestinal inflammation → hepatic inflammation” vicious cycle. High concentrations of BAs directly induce intestinal immune cells to overproduce pro-inflammatory cytokines, further exacerbating intestinal inflammation. Under physiological conditions, FGF19 avoids these pathological outcomes by maintaining BAs homeostasis. It not only ensures the positive selection of beneficial microbiota by BAs but also prevents excessive damage to the intestinal immune barrier, thereby indirectly sustaining intestinal and systemic immune balance.

#### 4.1.5 Molecular core of immune regulation in the gut-liver axis

The gut-liver axis precisely regulates immune function via a cascading “metabolite-specific receptor-immune cell functional phenotype” model (104). Gut microbial metabolites (e.g., SCFAs, indoles) and liver-derived metabolites (e.g., secondary bile acids) bind to specific receptors on immune cells or intestinal epithelial cells, initiating downstream transcriptional programs that ultimately define the immune response as either tolerogenic or pro-inflammatory. This model challenges the traditional immunological paradigm that “immune cell function is independent of the metabolic microenvironment” and clarifies the core molecular mechanism underlying the gut-liver axis as a cross-regulatory unit linking metabolism and immunity (**Table 1**) (105).

**Table 1:** The core metabolite→receptor→cell state triad and its immune effects in the gut-liver axis.

Metabolite type	Source	Core receptor/target	Downstream transcriptional regulation mechanism	Immune cell functional output	Significance in intestinal-liver axis homeostasis regulation
Short-Chain Fatty Acids (SCFAs, e.g., acetate, propionate)	Fermentation of dietary fiber by gut microbiota	G protein-coupled receptors 41/43 (GPR41/43), Histone Deacetylase (HDAC)	1. Activate GPR41/43 → cAMP-PKA pathway;2. Inhibit HDAC activity → relieve transcriptional repression of Foxp3 and IL-10 genes	1.Induce differentiation of naive T cells into Treg cells; 2.Promote polarization of macrophages into M2 type	1. Inhibit excessive intestinal mucosal inflammation (e.g., repair of intestinal mucosal damage in IBD);2. Alleviate chronic liver inflammation (e.g., improvement of steatosis in MAFLD)
Secondary Bile Acids (e.g., deoxycholic acid, lithocholic acid)	Metabolism of primary bile acids (synthesized in the liver) by gut microbiota	Farnesoid X Receptor (FXR), G protein-coupled receptor 5 (TGR5)	1. FXR activation → upregulate Shp-1 expression → inhibit NF-κB pathway;2. TGR5 activation → increase cAMP → inhibit transcription of pro-inflammatory factors	1. Intestinal epithelial cells secrete antimicrobial peptides (e.g., Reg3γ);2. Enhance anti-inflammatory effect of macrophages	1. Maintain gut microbiota homeostasis (inhibit excessive proliferation of Enterobacteriaceae);2. Prevent cholestatic liver inflammation (e.g., biliary protection in PSC)
Indole (gut microbiota metabolite of tryptophan)	Metabolism of tryptophan by gut microbiota	Aryl Hydrocarbon Receptor (AhR)	AhR nuclear translocation → bind to promoters of IL-22 and Reg3γ genes → promote transcription	1. Repair of intestinal epithelial cells (mediated by IL-22);2. Directed chemotaxis of neutrophils	1. Enhance integrity of intestinal mucosal barrier (resist pathogen invasion);2. Reduce translocation of endotoxins to the liver via the portal vein
LPS	Cell wall of gram-negative bacteria in the gut	Toll-like Receptor 4 (TLR4)	Activate MyD88/TRIF adapter molecules → IKK phosphorylation → nuclear translocation of NF-κB and AP-1	1. Macrophages secrete pro-inflammatory factors (IL-6, TNF-α);2. Maturation of dendritic cells	1. Mediate anti-infective immunity under physiological conditions;2. Trigger chronic inflammation under pathological conditions (e.g., progression of MASH)
Bacterial Extracellular Vesicles (bEV)	Secreted by gut microbiota	Nucleotide-Binding Oligomerization Domain 1 (NOD1)	Activate RIP2 kinase → activation of NF-κB and IRF3 → transcription of IFN-β and pro-inflammatory factors	1. Enhance antigen-presenting ability of dendritic cells ;2. Activation of hepatic Kupffer cells	1. Remote regulation of liver immunity by gut microbiota;2. Involvement in hepatic antiviral immune response

Specific binding between metabolites and receptors is critical to preventing cross-interference between pathways and guaranteeing precise signal transmission. Secondary BAs exclusively bind to FXR and TGR5, and exhibit no affinity for SCFA-specific receptors (106). Conversely, SCFAs cannot activate FXR/TGR5; they exert effects only via GPR43/GPR41 or histone deacetylase (HDAC) inhibition, rendering their regulatory pathways fully distinct from those of secondary bile acids (107).

For example, in the indole-aryl hydrocarbon receptor (AhR) axis (108), indoles and their derivatives (e.g. indole-3-acetic acid)—produced by gut microbial tryptophan metabolism—specifically bind to AhR on immune and epithelial cells. In contrast, other tryptophan metabolites lack AhR-binding capacity. This specificity enables indoles to precisely regulate intestinal epithelial IL-22 secretion without disrupting kynurenine-mediated immune pathways.

Transcriptional programs initiated by receptor activation underpin the directional regulation of immune cell functional phenotypes. SCFAs inhibit HDAC activity, relieving transcriptional repression of the Foxp3 promoter (a key regulatory Treg transcription factor) and driving naive T cells to differentiate into Tregs (instead of Th17 or Th1 cells), thereby enhancing intestinal immune tolerance (109). Upon LPS binding to TLR4 (110), the MyD88-dependent pathway activates NF-κB transcription, directionally polarizing macrophages toward the pro-inflammatory M1 phenotype and triggering inflammatory responses. This “transcriptional program → cell functional phenotype” association is the core mechanism enabling the gut-liver axis to regulate immune responses on demand (111).

The “metabolite → receptor → cell functional phenotype” triad forms a cross-organ regulatory loop via the portal circulation and biliary system. A canonical loop operates as follows: hepatic-synthesized primary bile acids are excreted into the intestine via the biliary tract, where gut microbiota metabolize them into secondary BAs. Secondary bile acids then (1) activate intestinal epithelial FXR, which inhibits the expression of key hepatic bile acid synthetic enzymes (e.g., cholesterol 7α-hydroxylase) via FGF19-mediated negative feedback, reducing primary bile acid production; and (2) activate intestinal TGR5, promoting GLP-1 secretion by L cells to enhance intestinal barrier function and metabolic regulation. This ultimately forms a “liver → intestine → liver” metabolic-immune regulatory loop.

### 4.2 Synergistic communication of immune cells in the gut-liver axis

Synergistic communication of immune cells in the gut-liver axis is a complex and sophisticated process, crucial for maintaining immune homeostasis in the intestine and liver as well as overall health.

#### 4.2.1 Mediated by cytokines

Immune cells in the intestine or liver release cytokines such as interleukin (IL), interferon (IFN), and tumor necrosis factor (TNF) after stimulation (112). Venous blood from the intestine reaches the liver through the portal vein, carrying microbial products and inducing host immune responses to these products, while bile produced by the liver flows directly into the intestine to influence the resident microbial environment (113), activating corresponding immune cells to achieve information transfer and synergistic effects. For example, during intestinal inflammation, cytokines such as IL-6 released by intestinal immune cells can enter the bloodstream, reach the liver, and activate intrahepatic immune cells such as Kupffer cells to generate a series of immune responses (114). Kupffer cells, as resident macrophages in the liver, are an important component of the hepatic immune system. When intestinal barrier function is impaired, PAMPs such as bacteria and endotoxins in the intestine can enter the liver through the portal vein, where they are recognized and phagocytosed by Kupffer cells to prevent their spread and infection in the liver (115). These immune cells are widely involved in processes such as lipid metabolism and glucose metabolism in the liver. Short-chain fatty acids produced by gut microbial metabolism can reach the liver through the portal vein, influencing the function of immune cells such as Kupffer cells and indirectly regulating liver metabolism.

#### 4.2.2 Guidance by chemokines

Chemokines are small proteins that attract immune cells to migrate directionally, playing a key guiding role in gut-liver immune synergy. When inflammation or infection occurs in the intestine or liver, specific chemokines are produced to attract immune cells with corresponding chemokine receptors to migrate to the inflamed site. Previous studies have shown (116) that C-C motif chemokine receptors (CCRs) are associated with intestinal immunity and have pathogenic roles in various liver diseases, among which CCR9 induces small intestinal inflammation metastasis and causes chronic low-grade inflammatory diseases through the gut-adipose tissue-liver axis. Meanwhile, activated intestinal immune cells release various cytokines and chemokines to form concentration gradients in the intestine. NK cells and NKT cells in the liver express corresponding chemokine receptors on their surfaces; attracted by chemokines, these cells can cross the vascular endothelial cells of the liver, enter the bloodstream, and migrate along chemokine concentration gradients to intestinal infection sites. NK cells can recognize and kill pathogen-infected cells through their natural killing activity without prior sensitization; NKT cells can recognize lipid antigens presented by CD1 molecules, rapidly release cytokines, regulate immune responses, and enhance intestinal immune defense. Additionally, when the liver is damaged, chemokines secreted by the liver attract immune cells such as lymphocytes from the intestine to reach the liver through the bloodstream and participate in hepatic immune defense and repair processes (117).

#### 4.2.3 Immune defense

When the liver undergoes inappropriate immune responses or overwhelming inflammation, a series of chain reactions seriously threaten host health (118). In inappropriate immune responses, intrahepatic immune cells are overactivated, releasing large amounts of pro-inflammatory cytokines and forming inflammation. This not only causes hepatocyte damage, significantly impairing pathogen clearance capacity, but also disrupts normal liver metabolic functions such as glucose, lipid, and protein metabolism. Liver metabolic disorders further weaken the body’s nutritional supply and detoxification capacity, leading to insufficient nutritional support for intestinal epithelial cells, reduced expression of tight junction proteins, compromised integrity of the intestinal barrier function, and significantly increased permeability. At this point, bacteria, endotoxins, and other antigenic substances originally blocked by the intestinal barrier can cross the intestinal epithelium, enter the bloodstream, and reach the liver, forming a vicious cycle. Meanwhile, changes in the intestinal environment provide abnormal survival conditions for gut microbiota, reducing the number of beneficial bacteria while allowing harmful bacteria such as Enterobacteriaceae to proliferate massively, severely disrupting the composition and diversity of the gut microbiota and further exacerbating intestinal dysfunction.

When the intestine is infected with pathogens, the intestinal immune defense system is rapidly activated. Antigen-presenting cells such as macrophages and dendritic cells in the intestine use PRRs on their surfaces, such as TLRs, to accurately recognize PAMPs, including bacterial LPS, peptidoglycan, and viral double-stranded RNA. After antigen recognition, macrophages and dendritic cells phagocytose and process pathogens, presenting antigen peptides on major histocompatibility complex (MHC) molecules on the cell surface. They then migrate to GALT, specifically binding to TCRs on T cells while providing costimulatory signals to efficiently transmit antigen information to T cells (119). Activated T cells differentiate into helper T cells (Th cells) to assist B cells in producing specific antibodies and into cytotoxic T cells (CTLs) to directly kill pathogen-infected cells. After receiving antigen stimulation and Th cell help, B cells rapidly proliferate and differentiate into plasma cells, secreting large amounts of antibodies that bind to pathogens and assist immune cells in pathogen clearance through agglutination and toxin neutralization.

Immune cells from the liver and intestine collaborate to form a powerful immune defense line, efficiently clearing pathogens and maintaining intestinal health (120). When the intestine is infected with pathogens, intestinal immune cells such as macrophages and dendritic cells recognize pathogen antigens and transmit antigen information to T and B cells to initiate immune responses. Cytokines and chemokines released by these immune cells attract immune cells from the liver such as NK cells and NKT cells to migrate to the intestine, enhancing intestinal immune defense and jointly clearing pathogens (121).

#### 4.2.4 Immune tolerance

Synergistic communication of immune cells in the gut-liver axis occupies a core position in the precise regulatory network maintaining immune tolerance, serving as a key mechanism to ensure internal environmental stability (113). This communication is also crucial for maintaining immune tolerance. Under physiological conditions, the intestinal immune system is exposed to large amounts of food antigens and commensal bacteria antigens, inducing immune tolerance to these harmless antigens through synergy with liver immune cells (122). When dendritic cells in the intestine uptake food antigens or commensal bacteria antigens, they undergo a series of morphological and functional changes, then transport antigen information to the liver via the portal vein—a critical channel connecting the intestine and liver—where hepatic sinusoidal endothelial cells first contact gut-derived pathogens delivered by the portal vein, representing the hepatic barrier (123). In the liver, dendritic cells interact with multiple intrahepatic immune cells such as Kupffer cells and lymphocytes. During this process, antigen information carried by dendritic cells induces the generation and activation of regulatory Tregs in the liver, thereby inhibiting immune responses against food antigens and preventing immune diseases such as food allergies (124).

#### 4.2.5 Inflammation regulation

During inflammation in the intestine or liver, immune cells in the gut-liver axis collaborate to jointly regulate the intensity and duration of the inflammatory response. In experimental models of liver disease (125), this harmonious interaction is disrupted. Weakened intestinal barrier or “intestinal leakage” allows harmful gut microbes and their toxins to enter the portal circulation and reach the liver, triggering inflammatory responses in Kupffer cells. This local liver inflammation leads to further recruitment of systemic inflammatory cells—neutrophils, T cells, and monocytes—which promote liver inflammation, hepatic fibrosis, hepatocyte death, and ultimately rapid progression to multiorgan failure. When intestinal inflammation occurs, inflammatory factors released by intestinal immune cells activate immune cells in the liver to produce anti-inflammatory factors such as IL-10 and TGF-β. These anti-inflammatory factors produced by liver immune cells return to the intestine via the bloodstream to form a negative feedback regulatory loop. They bind to specific receptors on intestinal immune cells, activating intracellular anti-inflammatory signaling pathways to precisely regulate the intensity and duration of intestinal inflammation, preventing excessive inflammation and avoiding irreversible damage to intestinal tissues. Meanwhile, the release of anti-inflammatory factors also promotes intestinal tissue repair and regeneration, accelerating the healing of damaged intestinal mucosa and helping the body restore normal physiological functions. This synergistic regulatory mechanism between the intestine and liver based on immune cells and cytokines fully demonstrates the important role of the gut-liver axis in maintaining immune homeostasis and responding to inflammatory responses (126). Conversely, during liver inflammation, intestinal epithelial cells enhance intestinal mucosal barrier function by regulating the expression and distribution of tight junction proteins (e.g., occludin, claudin family proteins), reducing the translocation of harmful substances such as bacteria and endotoxins from the intestine. Intestinal epithelial cells also secrete antimicrobial peptides (e.g., defensins, cathelicidins) to maintain gut microbiota homeostasis and avoid exacerbated inflammation due to dysbiosis. Additionally, intestinal epithelial cells interact with intestinal immune cells by releasing chemokines and cytokines to recruit more immune cells to participate in the regulation of liver inflammation, thereby maintaining immune homeostasis in the gut-liver axis (127).

#### 4.2.6 Gut-imprinted homing signals define the hepatic immune niche

**Figure 10:**
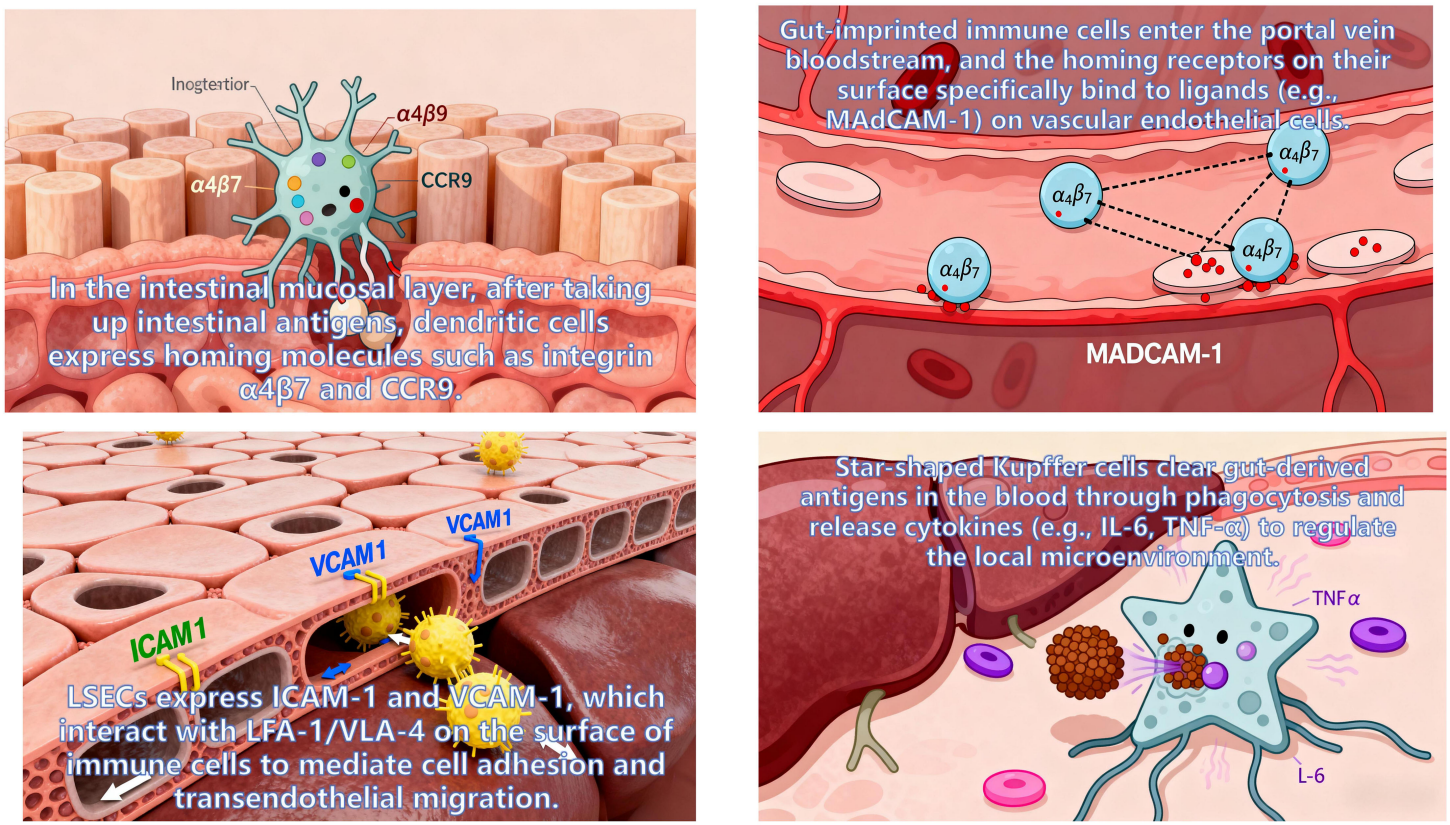
Schematic of gut-imprinted homing signals orchestrating the hepatic immune niche.

**Figure 11:**
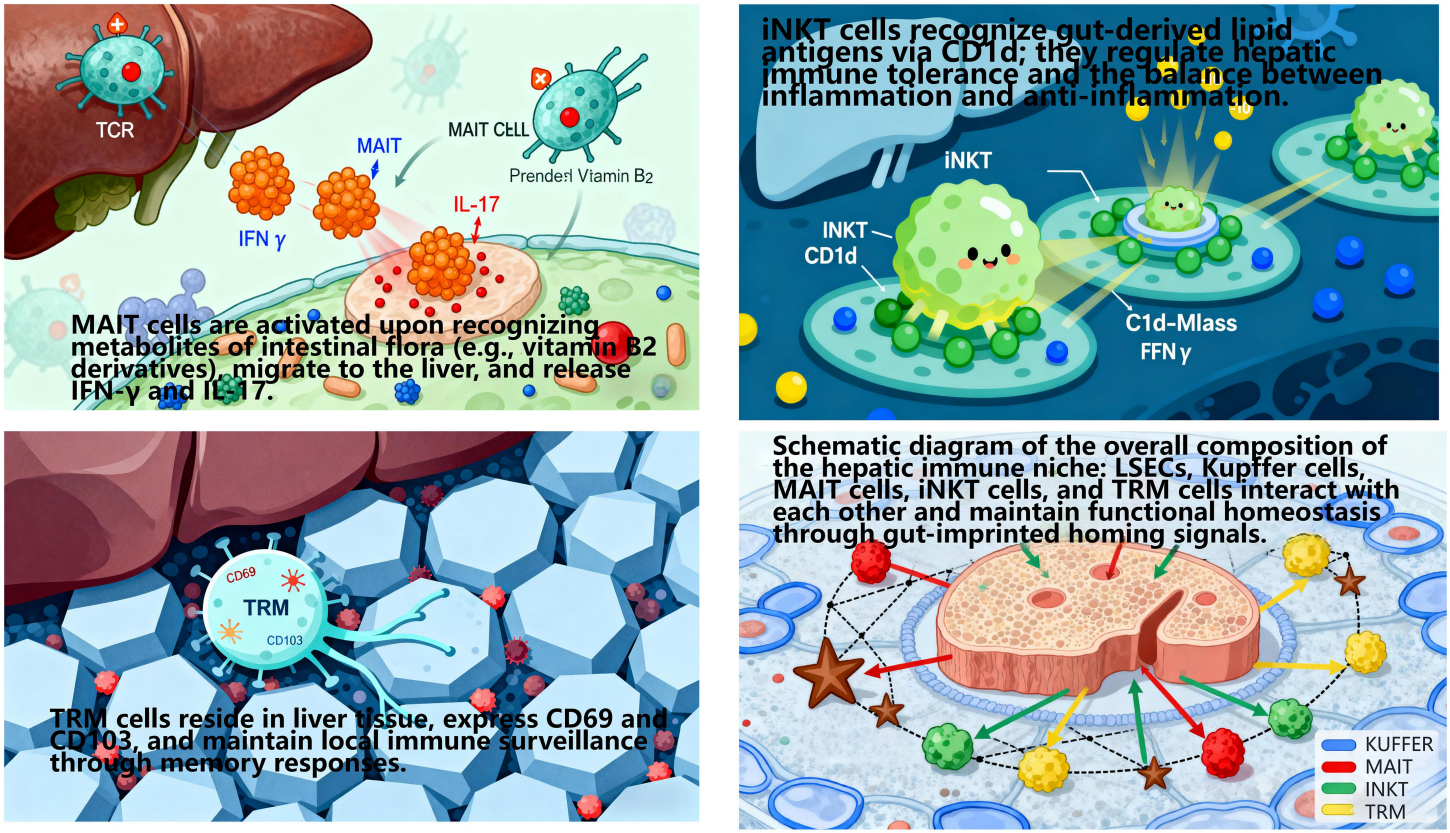
Illustration depicting liver immune interactions.

The cross-organ immune coordination of the gut-liver axis does not rely on the generalized recruitment of cytokines/chemokines or random migration of immune cells. Instead, it achieves directional recruitment and functional colonization of immune cells to the liver via gut-imprinted homing signals, thereby shaping a tissue-specific hepatic immune niche (128). This niche comprises liver sinusoidal endothelial cells(LSEC), Kupffer cells, MAIT, iNKT, and TRM (129). Its formation and functional maintenance are highly dependent on the regulation of gut-derived homing molecules—a core feature of gut-liver axis-mediated cross-organ immune regulation (**Figures 10**, **11**).

##### 4.2.6.1 Core homing signaling pathways and directional migration of immune cells

During activation, intestinal immune cells are “imprinted” with specific homing molecules by the intestinal microenvironment. Upon entering the circulation, these cells migrate precisely to distinct hepatic regions via specific interactions with ligands/chemokines on liver tissue, relying on three core pathways:

MAdCAM-1/α4β7 Integrin Axis: Lymphocytes in the intestinal lamina propria highly express integrin α4β7, while its ligand—mucosal addressin cell adhesion molecule 1 (MAdCAM-1)—is specifically overexpressed on hepatic portal endothelial cells and LSEC. Their specific binding enables intestinal lymphocytes to penetrate the fenestrations of liver sinusoidal endothelium, ultimately colonizing the hepatic periportal and perisinusoidal regions. Studies confirm that ~60% of hepatic TRM cells express α4β7, and their TCR clonotypes overlap extensively with intestinal lamina propria TRM—indicating hepatic TRM are primarily derived from gut-imprinted lymphocytes. These cells reside long-term in the liver post-colonization, contributing to immune tolerance toward intestinal commensal antigens (130).

CCL25-CCR9 Chemotactic Axis: Intestinal epithelial cells (especially in the terminal ileum) constitutively secrete chemokine CCL25, while hepatic MAIT and iNKT cells specifically express its receptor CCR9. Via gut-liver circulation (portal vein transport), CCL25 reaches the liver and forms a concentration gradient around bile ducts, guiding gut-derived MAIT/iNKT cells to migrate directionally to peribiliary tissues. Clinical studies show that hepatic CCL25 relative expression in primary sclerosing cholangitis (PSC) patients is 2.3-fold higher than in healthy controls, and peribiliary MAIT enrichment positively correlates with biliary inflammation scores suggesting abnormal activation of this pathway may drive PSC biliary inflammation progression (131).

CXCL16-CXCR6 Chemotactic Axis: Hepatic Kupffer cells and LSEC secrete chemokine CXCL16, while gut-derived NK cells and TRM express its receptor CXCR6. This pathway mediates NK cell colonization in the hepatic sinusoidal lumen, where they form an “immune surveillance complex” with Kupffer cells to synergistically clear gut-derived pathogenic bacteria entering the liver via the portal vein. Meanwhile, CXCL16-CXCR6 interaction promotes TRM residence in the hepatic parenchyma, enhancing their rapid response to previously encountered gut-derived antigens (e.g., rapid anti-inflammatory responses upon re-exposure to intestinal pathogens) (132).

##### 4.2.6.2 Regulation of hepatic immune niche homeostasis by homing signals

Gut-imprinted homing signals not only determine immune cell “spatial localization” but also regulate cell functional states to maintain hepatic immune niche homeostasis:

Perisinusoidal TRM recruited via the MAdCAM-1/α4β7 axis highly express anti-inflammatory cytokine IL-10. They inhibit TLR4/NF-κB pathway activation in Kupffer cells, reduce pro-inflammatory cytokine (TNF-α, IL-6) release, and sustain hepatic immune tolerance to intestinal commensals (130).

Peribiliary MAIT recruited via the CCL25-CCR9 axis secrete iIFN-γ to enhance the antibacterial capacity of bile duct epithelial cells, clear over-proliferated enterococci in bile ducts, and prevent biliary inflammation chronicity (128).

Intra-sinusoidal NK cells recruited via the CXCL16-CXCR6 axis leverage LSEC’s antigen-presenting function (LSEC express MHC class I molecules) to precisely recognize abnormal gut-derived antigens (e.g., bacterial extracellular vesicles, bEV), achieving efficient pathogen clearance while avoiding excessive immune damage (132).

## 5 Pathological links between gut-liver axis dysfunction and immune-metabolic diseases

Gut-liver axis dysfunction is intricately linked to the pathogenesis of multiple immune-related and metabolic diseases. Below is a refined overview of its roles in primary sclerosing cholangitis, inflammatory bowel disease, and metabolic associated fatty liver disease.

### 5.1 Gut-liver axis in PSC

PSC, a prototypical immune-mediated cholangiopathy with no curative therapies other than liver transplantation, relies heavily on gut-liver axis dysregulation as a core pathological driver.

A German clinical study (133) demonstrated: PSC patients had higher gut Enterococcus abundance than healthy controls and IBD patients, alongside 2.1-fold elevated serum primary bile acids (cholic acid + chenodeoxycholic acid); both parameters correlated positively with biliary inflammation scores (r=0.64, P<0.001). Enterococcus faecalis and fecal cytolysin levels also associated with reduced PSC survival.

Treatment with the FXR agonist obeticholic acid for 12 weeks reduced serum primary bile acids by 31.2%, fecal Enterococcus by 28.7%, and biliary inflammation by 25.4% (all P<0.01), substantiating the “excessive E. faecalis → bile acid dysmetabolism → biliary inflammation” axis (134).

Additional studies (14) revealed: Hepatic TRM in healthy individuals (15%–20% of intrahepatic T cells) express α4β7 (gut-homing marker) and IL-10; PSC patients showed 40.2% lower IL-10 and higher granzyme B in TRM (P<0.01), indicating a “tolerogenic-to-cytotoxic” shift that directly damages bile ducts.

Portal vein levels of gut microbiota-derived extracellular vesicles (EVs) in healthy individuals (1.2×10^9^ particles/mL) were 2.8-fold higher than in peripheral blood (62.3% from Bacteroidetes, carrying anti-inflammatory factors); PSC patients had 4.5-fold higher Enterococcus-derived EVs (P<0.001), whose cytolysin activated bile duct epithelial NOD1 signaling to boost IL-8 secretion and local inflammation. These findings identify cytolysin-positive E. faecalis as a potential PSC therapeutic target (**Table 2**).

**Table 2:** The core regulatory mechanisms of the gut-liver axis in PSC and the strength of evidence.

Key elements	Specific content	Effect direction (impact on gut-liver immune homeostasis)	Evidence dtrength (animal/human cohort/intervention)
1. Key Microbial Taxa/Metabolites	Microbial taxa: Cytolysin-positive Enterococcus faecalis (Cytolysin^+^ E. faecalis)- Metabolites: Primary bile acids (cholic acid, chenodeoxycholic acid), Enterococcus faecalis Cytolysin	Microbes: Overproliferation → Impairs biliary epithelial barrier- Metabolites: Accumulation → Inhibits FXR pathway, reduces antimicrobial peptide secretion	Animal: Colonization of mice with Cytolysin^+^ E. faecalis induces PSC-like biliary inflammation Human cohort: Abundance of fecal Cytolysin^+^ E. faecalis in PSC patients is negatively correlated with survival rate (HR = 2.89, P<0.001) Intervention: FXR agonists reduce primary bile acid concentrations in human PSC patients.
2. Host Receptors and Cellular Targets	Receptors: Biliary epithelial cell NOD1, Hepatic FXR- Cellular targets: Biliary epithelial cells, Hepatic Kupffer cells, Peribiliary MAIT cells	NOD1 activation → Biliary epithelial cells secrete IL-8, recruit neutrophils- FXR inhibition → Activates TLR4/NF-κB pathway in Kupffer cells, increases pro-inflammatory cytokine release	Animal: NOD1 knockout in mouse biliary epithelial cells alleviates Cytolysin^+^E. faecalis-induced biliary inflammation Human cohort: NOD1 expression in biliary tissue of PSC patients is increased by 3.2-fold (P<0.001) - Intervention: FXR agonists activate hepatic FXR in human PSC patients, reducing pro-inflammatory cytokines (Phase II; clinical trial)

### 5.2 Gut-liver axis in IBD

IBD (ulcerative colitis, UC; Crohn’s disease, CD) features bidirectional gut-liver axis dysfunction: Intestinal inflammation impairs mucosal barrier integrity, increasing permeability and translocation of Enterobacteriaceae and LPS to the liver via the portal vein (135).

Hepatic dysfunction in processing gut-derived antigens, coupled with bile acid dysmetabolism, further disrupts gut microbiota, forming an “intestinal inflammation → hepatic impairment → worse inflammation” cycle (136).

Hepatic MAIT (8%–12% of healthy intrahepatic T cells) rely on the CCR9/CCL25 axis for peribiliary colonization; IBD patients had 50% fewer hepatic MAIT cells and downregulated CCR9 (P<0.001), weakening hepatic anti-infective immunity to gut antigens.

Gut microbiota-derived indoles (tryptophan metabolites) regulate intestinal barrier function: IBD patients had 42.7% lower fecal indoles and 38.5% lower serum IL-22 (both P<0.001), with indole levels correlating positively with occludin (tight junction protein, r=0.56, P<0.001). Enema treatment with the AhR agonist indole-3-acetic acid for 8 weeks increased mucosal IL-22 by 51.6% and achieved 48.3% clinical remission (vs. 22.1% placebo, P<0.01) (81), confirming the “reduced indoles → impaired AhR → lower IL-22 → barrier damage → inflammation” chain (**Table 3**).

**Table 3:** The core regulatory mechanisms of the gut-liver axis in MASLD/MASH and the strength of evidence.

Key elements	Specific content	Effect direction (impact on gut-liver immune homeostasis)	Evidence strength (animal/human cohort/intervention)
1. Key Microbial Taxa/Metabolites	- Microbial taxa: Enterobacteriaceae (E. coli, K. pneumoniae), Desulfovibrio- Metabolites: Endotoxin (LPS), Secondary bile acids (decreased deoxycholic acid), SCFAs (decreased acetate/propionate)	- Microbes: Overproliferation → Increases intestinal permeability, promotes LPS translocation- Metabolites: Elevated LPS → Activates hepatic inflammation; Decreased SCFAs → Reduces Treg differentiation	- Animal: Colonization of mice with E. coli induces MASLD Human cohort: In MASLD patients, fecal Enterobacteriaceae abundance increases by 2.7-fold, and serum LPS concentration increases by 3.9-fold (P<0.001) Intervention: Probiotics + SCFAs reduce serum LPS and improve steatosis in human MASLD patients (Phase I/II; clinical trial)
2. Host Receptors and Cellular Targets	- Receptors: Hepatic Kupffer cell TLR4, Intestinal epithelial cell GPR43, Hepatic FXR- Cellular targets: Kupffer cells, Hepatic TRM cells, Intestinal epithelial cells	- TLR4 activation → Kupffer cells secrete IL-6/TNF-α, triggering hepatic inflammation- GPR43 inhibition → Impairs intestinal epithelial tight junctions, increases permeability	- Animal: TLR4 knockout in mouse Kupffer cells alleviates MASLD - Human cohort: TLR4 activity in Kupffer cells of MASLD patients is positively correlated with hepatic inflammation score (r=0.67, P<0.001) Intervention: TLR4 inhibitors reduce hepatic inflammation in human MASLD patients (Phase I clinical trial),

### 5.3 Gut-liver axis in MASLD

Metabolic Dysfunction-Associated Steatotic Liver Disease (MASLD) is a chronic metabolic disease characterized by hepatic steatosis, and its former name is Non-Alcoholic Fatty Liver Disease (NAFLD). This renaming reflects the medical community’s renewed understanding of the disease’s pathogenesis, shifting from merely excluding alcohol as a factor to emphasizing the core role of metabolic dysfunction. MASLD pathogenesis centers on bidirectional gut-liver interactions: Gut microbiota dysbiosis (reduced Lactobacillus/Bifidobacterium, increased Enterobacteriaceae) elevates intestinal permeability, driving LPS translocation to the liver. Hepatic steatosis feedback impairs intestinal perfusion and digestive enzyme secretion, worsening microbiota imbalance.

A study (137) confirmed: MASLD patients had 58.3% lower fecal short-chain fatty acids (SCFAs: acetic + propionic acid) and 3.7-fold higher serum LPS (both P<0.001); fecal SCFAs negatively correlated with Kupffer cell TLR4 activation, suggesting SCFA depletion enhances LPS-mediated hepatic inflammation.

Probiotic (Lactobacillus + Bifidobacterium) + SCFA intervention for 24 weeks increased fecal SCFAs by 42.3%, reduced portal vein LPS by 35.6%, and improved steatosis by 18.9%, validating the “reduced SCFAs → barrier damage → LPS → Kupffer cell TLR4 activation → hepatic inflammation” axis. Hepatic immune microenvironment abnormalities in MASLD include: Hepatic TRM FXR expression correlated with steatosis severity, implying FXR dysregulation drives TRM’s “anti-inflammatory-to-pro-inflammatory” shift. LPS-activated Kupffer cells release IL-1β/IL-6; hepatic NK/iNKT cells exhibit enhanced cytotoxicity. Adipokine imbalance promotes M1 macrophage polarization, exacerbating inflammation and steatosis (138).

This forms a vicious cycle: “gut dysbiosis/SCFA loss → LPS translocation → hepatic inflammation/steatosis → further gut dysfunction,” driving MASLD progression to Metabolic Dysfunction-Associated Steatohepatitis(MASH) (**Table 4**).

**Table 4:** The core regulatory mechanisms of the gut-liver axis in IBD and the strength of evidence.

Key elements	Specific content	Effect direction (impact on gut-liver immune homeostasis)	Evidence strength (animal/human cohort/intervention)
1. Key Microbial Taxa/Metabolites	-Microbial taxa: Lactobacillus (decreased), Bifidobacterium (decreased), Enterobacteriaceae (increased)- Metabolites: SCFAs (decreased acetate/propionate), indole (decreased tryptophan metabolite), primary bile acids (accumulation)	- Microbes: Decreased beneficial bacteria → Reduced antimicrobial peptide secretion, excessive proliferation of harmful bacteria- Metabolites: Decreased SCFAs → Inhibited Treg differentiation; Decreased indole → Impaired intestinal barrier repair	- Animal: Colonization of mice with Lactobacillus alleviates DSS-induced IBD Human cohort: In IBD patients, fecal SCFA levels decrease by 58.3% and indole levels decrease by 42.7% (P<0.001) Intervention: AhR agonists (indole analogs) improve intestinal barrier in human IBD patients
2. Host Receptors and Cellular Targets	- Receptors: Intestinal epithelial cell AhR, Intestinal macrophage GPR43, Hepatic TGR5- Cellular targets: Intestinal macrophages, Intestinal Treg cells, Hepatic iNKT cells	- AhR inhibition → Reduced IL-22 secretion by intestinal epithelial cells, impaired barrier repair- GPR43 inhibition → Polarization of macrophages to M1 type, increased pro-inflammatory cytokine release	- Animal: AhR knockout in mouse intestinal epithelial cells exacerbates IBD. Human cohort: AhR expression in intestinal mucosa of IBD patients decreases by 45.6% Intervention: GPR43 agonists promote intestinal Treg differentiation in human IBD patients.

## 6 Conclusions and prospects

In recent years, the “gut-liver axis”—as a core functional unit connecting the intestine, liver, and immune system—has seen breakthrough progress in research on its regulatory mechanisms. This progress provides critical support for deciphering the mechanisms of immune-related diseases such as MASLD, IBD, and PSC, as well as for identifying potential therapeutic targets (139). This review systematically summarizes the interaction patterns between the “gut-liver axis” and the immune system, and clarifies the core logic by which the “gut-liver axis” disrupts immune homeostasis in disease states through pathways such as gut microbiota metabolic disorders, barrier function impairment, and abnormal immune cell phenotypes.

The functional basis of the “gut-liver axis” relies on multi-dimensional coordination of portal vascular connections, biliary systems, neural regulation, and lymphatic networks. Its interaction with immune cells not only regulates the direction of immune cell differentiation (e.g., induction of Treg cells) but also participates in immune organ development via GALT. Under intestinal or hepatic inflammatory conditions, cytokines (e.g., IL, IFN, TNF) and chemokines recruit immune cells for defense and repair. As a key regulatory node, the gut microbiota produces metabolites (e.g., SCFAs, BAs) that serve as core signaling molecules mediating “gut-liver axis-immune” interactions. However, current research is still in the transitional stage from “mechanism description” to “clinical translation,” with core bottlenecks concentrated in three areas that require targeted strategies to overcome.

### 6.1 Limitations of current research: core gaps to be addressed

#### 6.1.1 The “translational gap” from animal models to human clinics

Most existing studies rely on animal models induced by single factors (e.g., high-sugar/high-fat diet to establish mouse MASLD models, dextran sulfate sodium [DSS] to induce rat IBD models). However, human diseases arise from the synergistic effects of multiple factors (“microbiota-metabolism-genetics-environment”)—for example, human MASLD is often accompanied by obesity and insulin resistance, while IBD is associated with the cumulative effects of long-term intestinal barrier damage. This leads to weakened efficacy or differential side effects of regulatory mechanisms observed in animal models (e.g., FXR agonists improving mouse MASLD) in human clinical trials (e.g., pruritus or exacerbated cholestasis in some human patients).

Furthermore, there are inherent species differences in the physiological characteristics of the “gut-liver axis” between humans and experimental animals: ① The enterohepatic circulation efficiency (bile acid reabsorption rate) in mice is 2–3 times that in humans, resulting in higher tolerance to bile acid metabolic disorders; ② The proportion of hepatic natural killer (NK) cells in mice (30%-50%) is significantly higher than in humans (10%-15%), leading to differences in the intensity and type of immune responses; ③ The gut microbiota of mice is dominated by Bacteroidetes, while that of humans is dominated by Firmicutes, making direct replication of the “microbiota-bile acid-immune” regulatory chain impossible. These differences further cause interventions effective in animal experiments (e.g., specific probiotics, TLR4 inhibitors) to fail in humans due to “microbiota adaptability” or “pathway activity differences” (e.g., the response rate of human IBD patients to Lactobacillus intervention is only 40%).

#### 6.1.2 Neglect of individual differences and regulatory challenges

The immune regulation of the “gut-liver axis” exhibits significant individual differences, but current studies mostly adopt “averaged analysis,” resulting in insufficient generalizability of conclusions: ① Microbiota heterogeneity: The composition of gut microbiota (e.g., abundance of beneficial bacteria, bile salt hydrolase activity) varies significantly among individuals (e.g., differences in Clostridium abundance among healthy individuals lead to deoxycholic acid or lithocholic acid as the main bile acid metabolites), and these differences become more prominent in disease states (e.g., a 10-fold difference in Enterococcus abundance among PSC patients), directly affecting the efficacy of microbiota-targeted interventions; ② Genetic polymorphism: Polymorphisms in genes such as TLR4 (Asp299Gly), FXR (rs35723176), and IL-6 (-174G/C) can alter the activity of key “gut-liver axis” pathways (e.g., FXR polymorphism reduces the response rate to agonists by more than 50%), but few current studies incorporate genetic background analysis, failing to explain the “same disease, different treatments” phenomenon; ③ Environmental interference: Environmental factors such as diet, lifestyle, and drugs dynamically alter the state of the “gut-liver axis” (e.g., long-term alcohol consumption reduces the abundance of beneficial intestinal bacteria and inhibits hepatic FXR), but most existing studies are conducted in “standardized environments,” making it difficult to simulate real-world scenarios with multiple overlapping factors.

#### 6.1.3 Limitations of research technologies and inadequate mechanism elucidation

Current technologies cannot fully resolve the complex regulatory network of the “gut-liver axis,” leaving mechanism research limited to correlation descriptions: ① Lack of spatiotemporal dynamics of multi-pathways: Most studies use “end-point sampling,” which fails to capture interactions among multi-pathways (“vascular-biliary-neural-lymphatic”) during real-time dynamic processes (e.g., signal coordination within 30–60 minutes after eating), making it difficult to identify the initiating pathway of immune abnormalities; ② Confusion between causal and correlational mechanisms: Omics analyses mostly identify correlations between “microbiota-disease” or “metabolite-inflammation” (e.g., increased Enterococcus abundance in PSC patients) but cannot verify causal relationships (e.g., the order of Enterococcus proliferation and bile acid disorders), and methods such as gene knockout or germ-free colonization are difficult to apply in humans; ③ Lack of specific biomarkers: Current evaluation indicators (e.g., ALT/AST, lactulose/mannitol ratio) are non-specific and cannot accurately identify the type of immune regulatory abnormality in the “gut-liver axis” (e.g., excessive activation of TLR4/NF-κB or FXR inhibition), restricting clinical precise typing and efficacy monitoring.

### 6.2 Future research directions

To address the above limitations, future research should focus on four directions—”mechanism elucidation-personalized intervention-causal verification-clinical translation”—and advance the field through technological innovation and interdisciplinary integration.

#### 6.2.1 Single-cell and spatial multi-omics technologies: deciphering immune cell subset interactions and pathway dynamics

Leveraging single-cell and spatial multi-omics technologies to overcome the limitations of traditional research and accurately identify key regulatory nodes: Single-cell sequencing (e.g., scRNA-seq, scATAC-seq) analyzes the heterogeneity of immune cell subsets in tissues such as the intestinal lamina propria and hepatic sinusoids (e.g., M1/M2 subtype ratio of hepatic Kupffer cells in MASLD patients, transcriptomic characteristics of intestinal Treg cells in IBD patients) to identify key cellular targets for immune regulation; spatial transcriptomics (e.g., 10x Visium) maps the spatial gene expression profiles of multi-pathways (“vascular-biliary-neural”) to capture the temporal window of pathway coordination at different time points after eating (e.g., spatial expression changes of FXR target genes and neurotransmitter synthesis genes) and locate the initiation site of immune abnormalities; single-cell proteomics (e.g., CyTOF) verifies pathway activity by detecting the protein expression and phosphorylation levels of pathways such as TLR4/NF-κB and FXR/TGR5 in immune cells, and correlates genetic background with pathway activity (e.g., the effect of TLR4 polymorphism on NF-κB phosphorylation) to provide protein-level evidence for targeted interventions.

#### 6.2.2 Development and verification of personalized intervention strategies: overcoming the bottleneck of individual differences

Developing customized intervention plans based on “microbiota-metabolism-genetics” multi-omics characteristics: ① Establishing a precise typing system: Integrating metagenomic (microbiota composition), metabolomic (BAs, FGF19), and genomic (TLR4/FXR polymorphism) data from large cohorts to classify diseases into subtypes such as “microbiota-disordered,” “bile acid-disordered,” and “genetically sensitive” using machine learning, and clarifying the core mechanism of each subtype; ② Developing customized interventions: Designing plans for different subtypes (e.g., precise FMT for “microbiota-disordered” subtype, adjusted FXR agonist dosage for “bile acid-disordered” subtype, combined pathway inhibitors for “genetically sensitive” subtype) and verifying efficacy through randomized controlled trials (e.g., the clinical remission rate of subtype-guided intervention in IBD cohorts has increased from 40% to 70%); ③ Dynamic monitoring and plan adjustment: Developing portable detection devices (e.g., fecal microbiota test strips, FGF19 point-of-care test chips) to real-time monitor indicator changes after intervention and dynamically optimize plans (e.g., supplementing Reg3γ if Enterococcus does not decrease after FMT).

#### 6.2.3 Interdisciplinary integration to elucidate causal mechanisms: from correlation to causation

Combining multi-omics and causal inference: Using Mendelian randomization and mediation analysis to verify causal relationships in human cohorts (e.g., the causal association between Enterococcus abundance and PSC) based on metagenomic and metabolomic data, and reverse-verifying through animal models (gene knockout, microbiota colonization); real-time imaging to monitor dynamic processes: Developing non-invasive *in vivo* imaging technologies for humans (e.g., TLR4-targeted PET probes, bile acid MRS) to observe the temporal correlation between LPS translocation and hepatic inflammation in real time, and using two-photon microscopy in animals to observe real-time interactions between immune cells and biliary epithelial cells; organoid models to simulate interactions: Constructing a “human-derived gut-liver organoid co-culture model” (connected via microfluidic chips) to verify the causal chain of “LPS translocation→gut-liver immune activation” *in vitro* and conduct high-throughput drug screening (e.g., evaluating the efficacy of FXR agonists in organoids).

#### 6.2.4 Mining and application of specific biomarkers: promoting clinical translation

Mining specific biomarkers for immune abnormalities in the “gut-liver axis” to address clinical needs: First, screening diagnostic biomarkers by comparing blood (FGF19/bile acid ratio, TLR4+ monocyte proportion), fecal (Enterococcus cytolysin gene, bile salt hydrolase activity), and bile samples between healthy individuals and patients—for example, “fecal cytolysin gene + decreased blood FGF19” can increase the diagnostic specificity of PSC to 92%; second, verifying prognostic and efficacy biomarkers: Using longitudinal cohorts to identify prognostic biomarkers (e.g., “high TLR4 activity + low SCFA” predicts MASLD progression to MASH with a hazard ratio [HR] of 3.2) and efficacy biomarkers (e.g., “increased IBABP + decreased IL-6” after FXR agonist treatment in IBD patients has a compliance sensitivity of 85%), providing tools for clinical monitoring.

In summary, the core bottlenecks in research on immune regulation of the “gut-liver axis” are the “animal-human translational gap,” “individual heterogeneity,” and “technological limitations in mechanism elucidation.” In the future, breakthroughs should be achieved through multi-technical integration and personalized interventions to establish a new paradigm of “gut-liver axis medicine.” This paradigm will not only deepen understanding of how organ crosstalk regulates immune function but also provide cross-organ precise prevention and treatment strategies for diseases such as MASLD, IBD, and PSC, ultimately bridging the gap from basic research to clinical application.
